# The Diversity of Chemoprotective Glucosinolates in Moringaceae (*Moringa* spp.)

**DOI:** 10.1038/s41598-018-26058-4

**Published:** 2018-05-22

**Authors:** Jed W. Fahey, Mark E. Olson, Katherine K. Stephenson, Kristina L. Wade, Gwen M. Chodur, David Odee, Wasif Nouman, Michael Massiah, Jesse Alt, Patricia A. Egner, Walter C. Hubbard

**Affiliations:** 1 Cullman Chemoprotection Center, Johns Hopkins University, Baltimore, Maryland USA; 2 Johns Hopkins University School of Medicine, Department of Medicine, Division of Clinical Pharmacology, Baltimore, Maryland USA; 3 Johns Hopkins University School of Medicine, Department of Pharmacology and Molecular Sciences, Baltimore, Maryland USA; 4 Johns Hopkins University Bloomberg School of Public Health, Department of International Health, Center for Human Nutrition, Baltimore, Maryland USA; 5 Instituto de Biología, Universidad Nacional Autónoma de México, Tercer Circuito de Ciudad Universitaria, Ciudad de México, 04510 Mexico; 6 The International Moringa Germplasm Collection, Ejido de la Reforma Agraria, Jalisco Mexico; 7 Biotechnology Laboratory, Kenya Forestry Research Institute, Nairobi, Kenya; 8 Department of Forestry, Range, and Wildlife Management, Bahauddin Zakariya University, Multan, Pakistan; 9 George Washington University, Department of Chemistry, Columbian College of Arts and Sciences, Washington DC, USA; 10 Johns Hopkins Drug Discovery, Baltimore, Maryland USA; 11 Johns Hopkins University Bloomberg School of Public Health, Department of Environmental Health and Engineering, Baltimore, Maryland USA; 12 Present Address: Graduate Group in Nutritional Biology, UC Davis, Davis, California, USA

## Abstract

Glucosinolates (GS) are metabolized to isothiocyanates that may enhance human healthspan by protecting against a variety of chronic diseases. *Moringa oleifera*, the drumstick tree, produces unique GS but little is known about GS variation within *M*. *oleifera*, and even less in the 12 other *Moringa* species, some of which are very rare. We assess leaf, seed, stem, and leaf gland exudate GS content of 12 of the 13 known *Moringa* species. We describe 2 previously unidentified GS as major components of 6 species, reporting on the presence of simple alkyl GS in 4 species, which are dominant in *M*. *longituba*. We document potent chemoprotective potential in 11 of 12 species, and measure the cytoprotective activity of 6 purified GS in several cell lines. Some of the unique GS rank with the most powerful known inducers of the phase 2 cytoprotective response. Although extracts of most species induced a robust phase 2 cytoprotective response in cultured cells, one was very low (*M*. *longituba*), and by far the highest was *M*. *arborea*, a very rare and poorly known species. Our results underscore the importance of *Moringa* as a chemoprotective resource and the need to survey and conserve its interspecific diversity.

## Introduction

Glucosinolates (GS) account in part for the remarkable medicinal potential of *Moringa oleifera* (“moringa,” Moringaceae, Brassicales; apparently native to the sub-Himalayan lowlands in NW India)^1^. Glucosinolates (β-thioglucoside *N*-hydroxysulfates), mostly restricted to the angiosperm order Brassicales^2^, are metabolized by the enzyme myrosinase to their biologically active, cognate isothiocyanates (ITC)^1,3,4^. Isothiocyanates have long been known for their herbivore deterrent, fungicidal, bacteriocidal, nematocidal, and allelopathic properties^5–11^. Isothiocyanates such as sulforaphane from broccoli have antibiotic activity against numerous human pathogens including *Escherichia coli*, *Salmonella typhimurium*, *Candida* sp., and *Helicobacter pylori*^12–18^. These medicinal properties have been ascribed both to temperate cruciferous plants that are well-known sources of glucosinolates, and to *Moringa oleifera*, the most widely cultivated and economically important species of the monogeneric tropical family Moringaceae^19–22^. Because moringa is highly drought resistant, it can provide benefits to the large and often underserved human populations in the tropics and sub-tropics worldwide.

Many of the medicinal properties such as cancer treatment, regulation of blood glucose levels, and antibiosis that have long been ascribed to *M*. *oleifera* in traditional medicine are likely attributable to its glucosinolates or isothiocyanates^22^. For example, one of the main uses of *M*. *oleifera* in Ayurvedic tradition is cancer treatment^23^. The biomedical research literature now contains numerous animal studies showing preventive effects against carcinogenesis^22^ that are plausibly accounted for by known mechanisms of action of non-moringa GS and ITC^7–25^. Additionally, we showed that 4-α-L-rhamnopyranosyloxy)benzyl isothiocyanate (4RBITC), the isothiocyanate created by hydrolysis of “glucomoringin” (4RBGS or 4-(α-L-rhamnopyranosyloxy)benzyl glucosinolate) from *M*. *oleifera* is a potent and selective antibiotic against *H*. *pylori*^15^. Other studies have shown that the antibiotic activity of 4RBITC from *M*. *oleifera* is selective and potent against other important human pathogens such as *Staphylococcus aureus* and *Candida albicans*^26^. It also appears to be effective in controlling certain manifestations of both ALS and multiple sclerosis in mouse models^27^^,^^28^. A growing number of epidemiologic, animal, and clinical studies link dietary glucosinolates and their cognate isothiocyanates to protection against chronic diseases including a variety of cancers, diabetes, and autism spectrum disorder via the Keap1-Nrf2-ARE-mediated induction of phase 2 cytoprotective enzymes^29–44^. The coordinated Nrf2-mediated upregulation of this large group of enzymes is responsible for the very important indirect antioxidant activity of these isothiocyanates^31,34,45–50^.

It is likely that the chemistry of the uniquely rhamnosylated glucosinolates from *Moringa* spp. (compared to all 120 or so other glucosinolates) might provide special advantages to mammals consuming them at moderate levels^22,37^. Outside Moringaceae, rhamnosylated glucosinolates have only been documented in the related Resdaceae (*Reseda* spp.)^51,52^ and Brassicaceae (*Noccaea caerulescens*)^53^. One reason for particular interest in the rhamnosylated glucosinolates is the strong possibility of biologically significantly different absorption, distribution, metabolism, and excretion compared to other glucosinolates^2,22,43,54^.

With these considerations in mind, we screened glucosinolate diversity and phase 2 enzyme induction potential across *Moringa*. We focus primarily on adult leaves, because in *M*. *oleifera* and *M*. *stenopetala* leaves are the most commonly used parts of the plant, providing nutritious vegetables that are consumed both fresh and cooked. We also examine young leaves and the exudates from leaf glands of some plants. Glands on young leaves secrete a clear, sticky exudate that often attracts ants^55^. Because glucosinolates, via their cognate isothiocyanates, serve anti-herbivory and other protective functions, these compounds are often present in highest quantities at early, more vulnerable ontogenetic stages^56,57^. Young leaves, mature leaves, seeds, flowers, and extrafloral nectaries might all be expected to have differing proportions of glucosinolates. Our study thus provides a survey of the diversity and relative amounts of glucosinolates across the leaves, seeds, and exudates across this small but chemically and morphologically diverse family.

To the extent that morphological diversity reflects potentially different ways of interacting with herbivores, it is reasonable to presume that surveying across *Moringa* species could identify species with compounds that are even more efficacious than those currently known. In addition to the commonly grown *M*. *oleifera*, there are 12 other species in this monogeneric family. All of the species are native to the dry tropics of Africa, Asia, and Madagascar, with the center of diversity being the Horn of Africa at the intersection of Kenya, Ethiopia, and Somalia. The most commonly cultivated species *M*. *oleifera* is apparently native to northwestern Indian lowlands, and seems likely to have been domesticated in India thousands of years ago, with the domesticate differing markedly from the wild plants in its much faster growth rate, shorter maturation time, and softer leaflets. Its close relative *M*. *concanensis* is also native to the Indian subcontinent, where it is relatively widespread in dry tropical woodlands. The closest relative to the Indian species is *M*. *peregrina*, which is found from the Dead Sea south to the northern Horn of Africa. Four species with massive, water-storing trunks are found in Madagascar (*M*. *drouhardii* and *M*. *hildebrandtii*), Namibia and Angola (*M*. *ovalifolia*), and Kenya and Ethiopia (*M*. *stenopetala*). A group of closely-related species is restricted to the Horn of Africa, and is made up of medium sized to small trees with tuberous roots (*M*. *arborea*, *M*. *rivae*, *M*. *ruspoliana*), to dwarf shrubs to tiny herbs with massive underground tubers (*M*. *borziana*, *M*. *longituba*, *M*. *pygmaea*). Sampling across the diversity from massive trees to tiny herbs, we explore the diversity of glucosinolates in leaves, seeds, bark, inflorescences, and glandular exudates from both field and cultivated specimens of 12 of the 13 species.

We assess the glucosinolate contents of 12 of the 13 known species of *Moringa*, mostly in leaves (dried and fresh), but also when available seeds, stems, and leaf gland exudates. We document the occurrence of 2 heretofore unidentified glucosinolates as major components of 6 *Moringa* species, though they are not abundant in the 2 most common species (domestic *M*. *oleifera* and *M*. *stenopetala*). We report on the occurrence of simple alkyl glucosinolates in four species (*M*. *peregrina*, *M*. *ruspoliana*, *M*. *rivae*, and *M*. *longituba*), in one of which (*M*. *longituba*) they are the dominant glucosinolates. Finally, we document potent chemoprotective potential in most of the species, show that the activity comes from their glucosinolates, and measure the cytoprotective activity of 6 of these glucosinolates in purified form in a variety of cell lines. We could find no obvious relationships between growth habit, geography, phylogenetic relatedness or other obvious variables, and glucosinolate content or spectrum, underscoring the importance of conserving and studying all the species in the genus.

## Results

We evaluated the major GS in leaf samples from 14 *Moringa* taxa, including 12 of the 13 known wild *Moringa* species, plus samples from domesticated *M*. *oleifera* as well as a hybrid between *M*. *concanensis* and *M*. *oleifera*. Given availability, our sampling also included samples from seeds of 9 taxa, leaf gland exudate from 6, bark from one species, and inflorescence axis samples from another. All GS were initially separated by HPLC. Peaks were only purified and/or collected for mass spectrometry (MS) and nuclear magnetic resonance spectrometry (NMR) if the matching peak from a second otherwise identical extract disappeared after enzyme treatment with myrosinase, which is highly specific for GS. While confirming the presence of some compounds, our sampling revealed a rich diversity of compounds and concentrations across species, including two apparently newly identified GS. Associated with this diversity was a very wide range of potency of induction of the phase 2 detoxication response in mammalian cells, including some responses that were very high, surpassing that of the well-characterized isothiocyanates such as sulforaphane.

**Figure 1:**
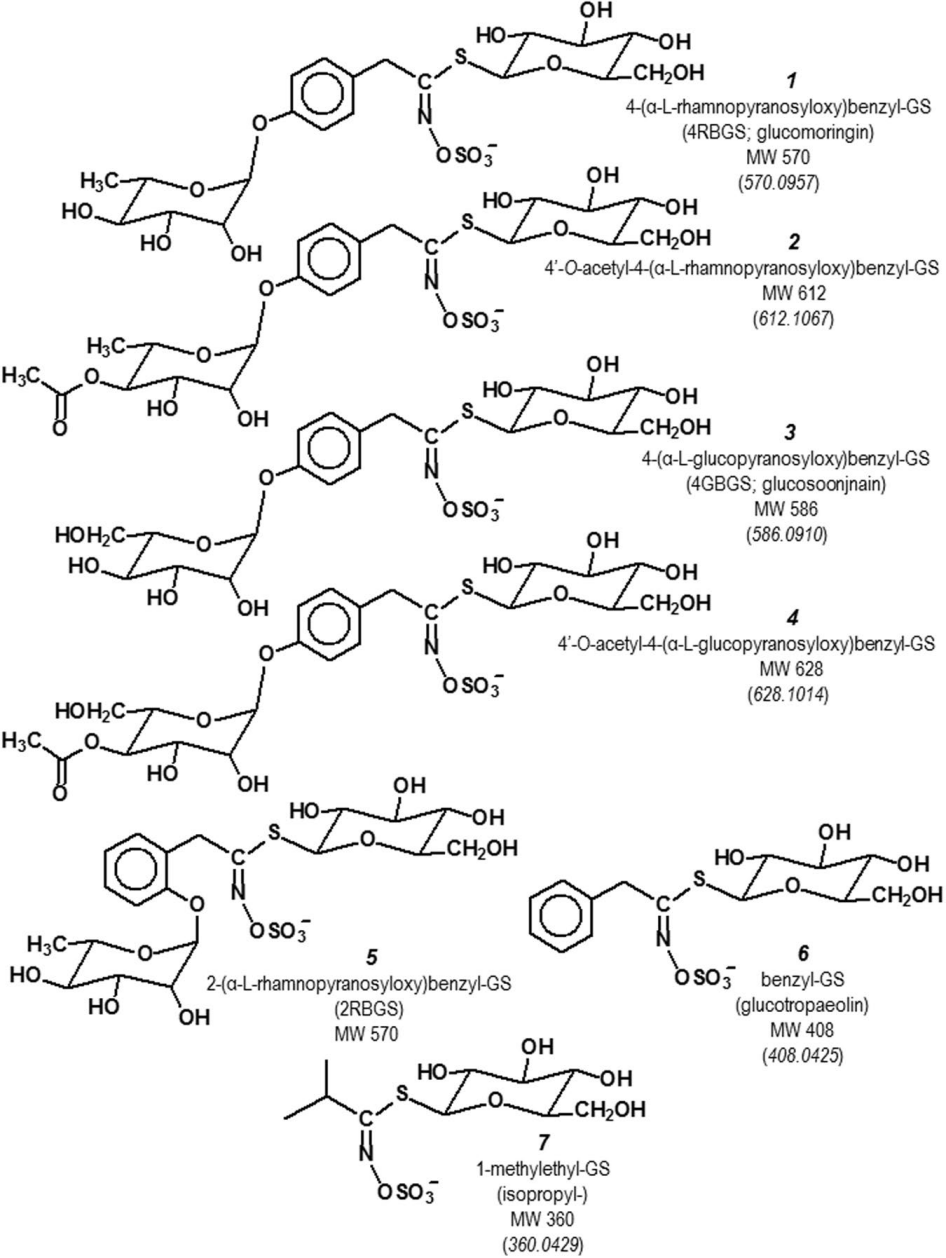
The predominant glucosinolates (GS) in the genus Moringa were isolated from plant organ extracts of the 12 *Moringa* spp. described herein, by complementary HPLC techniques, and identities were confirmed initially by direct mass spectroscopy (*m/z*), and by MS/MS. Accurate mass determination is given in parentheses under the nominal mass designation. NMR was utilized to confirm the identities of compounds ***1*** and ***2*** which are described in the literature, and of ***3*** and ***4*** which to our knowledge have heretofore not been described. GS ***5*** is not found in *Moringa* spp. but is an isomer of ***1*** that we isolated from *Reseda odorata* and provide by way of comparison. The biological activities of ITCs generated from these GS are presented in Table 1.

We confirmed the presence of the previously identified rhamnose-containing glucosinolate (4RBGS ***1*** *m/z* 570.0957) and its acetylated derivatives (e.g. ***2*** *m/z* 612.1067) as the predominant GS in *Moringa oleifera* domestic and *M*. *stenopetala* (Fig. 1). Also in these two species, we identified two multiply glycosylated GS which to our knowledge have never before been described. They are shown as ***3*** & ***4*** in Fig. 1. They contain a glucopyranosyloxy-benzyl moiety as their R-group (not described previously), as opposed to the rhamnopyranosyloxy-benzyl moiety that has been previously reported in GS from *Moringa* spp. Upon further investigation, we found this glucopyranosyloxy-benzyl GS (***3***) in all *Moringa* species except *M*. *hildebrandtii*. We found the former, well-documented rhamnopyranosyloxy-benzyl GS (***1***) in all species except *M*. *longituba*.

We obtained unequivocal structural identification of these compounds from nominal and accurate masses via electrospray MS, from LC/MS/MS (*m/z* 586.0900 and *m/z* 628.1014 for compounds ***3*** and ***4*** respectively), and by 400 and 600 MHz NMR according to methods described previously^58^ (Supplemental Data & Supplemental Fig. S1).

Given nomenclatural disagreements in the literature and configurational differences (e.g. isomers that are acetylated at the 2′ or 3′ position on the side-chain sugar (rhamnose or glucose), rather than at the 4′ position we have indicated, we refer in the ensuing presentation of data only to “monoacetyl-4-(α-L-rhamnopyranosyloxy)benzyl-GS” or “monoacetyl-4-(α-L-glucopyranosyloxy)benzyl-GS” for the sake of simplicity and accuracy. In Fig. 1, we show only 4′-*O*-acetyl-4-(α-L-rhamnopyranosyloxy)benzyl GS (***2***) and 4′-*O*-acetyl-4-(α-L-glucopyranosyloxy)benzyl GS (***4***). In all of our samples the molecular weights of compounds ***2*** and ***4***, the acetylated derivatives of compounds ***1*** and ***3*** respectively, were consistent with the identities provided.Other GS were identified in *Moringa* spp. that were consistent with those described previously^52,59,60^. Identities were matched with authentic standards where possible and accurate masses were obtained by mass spectroscopy of HPLC peaks. As noted above NMR confirmation was also obtained with compounds ***1***, ***3***, and ***4***. Thus, in addition to the GS noted above, we identified benzyl GS ***6***, and 1-methylethyl-GS ***7***. These GS are all shown in Fig. 1. The final GS found in the 12 *Moringa* species examined was not unequivocally identified and has been referred to in Fig. 1 as “alkyl-GS”. This compound or compounds has an accurate mass of 390.0534, which is consistent with 5 different isomeric GS: 3-hydroxybutyl GS, 4-hydroxybutyl GS, 1-(hydroxymethyl)propyl GS, 2-hydroxy-2-methylpropyl GS, and 1-ethyl-2-hydroxyethyl GS^2^. This GS was only found in *M*. *longituba*, a dwarf species with red, tubular flowers unique in Moringaceae, from Kenya, Ethiopia, and Somalia.

We also have included a non-*Moringa* GS in Fig. 1 for comparison. It is a benzyl GS that is rhamnosylated at the *ortho*- or 2- position on the benzyl ring (2-(α-L-rhamnopyranosyloxy)benzyl GS; ***5***). We have isolated this compound from *Reseda odorata*, which like *Moringa* is a member of Brassicales. The purified compound a structural isomer of compound ***1*** from *Moringa* in which the rhamnose group attaches at the 4- or para- position of the benzyl ring, is a much less potent inducer of cytoprotective enzyme than compound ***1***, as shown in Table 1.

**Table 1:** Phase 2 enzyme inducer potencies of selected isothiocyanates (ITC) produced by myrosinase hydrolysis of purified glucosinolates (GS).

GS^a^	Isothiocyanate (ITC)	Source	NQO1^b^				GSH^c^
			Hepa	RAW	PE	ARPE	Hepa
*1*	4-(α-L-rhamnosyloxy)benzyl-ITC	*M*. *oleifera*	0.05	2.3	1.2	3.0	1.8
*2*	monoacetyl-4-(α-L-rhamnosyloxy)benzyl-ITC	*M*. *oleifera*	0.6	2.3	3.7	>10	2.2
*3*	4-(α-L-glucosyloxy)benzyl-ITC	*M*. *borziana*	1.0	4.5	3.9	>10	4.0
*4*	monoacetyl-4-(α-L-glucosyloxy)benzyl-ITC	*M*. *borziana*	0.4	1.9	1.9	>10	1.6
*5* ^d^	2-(α-L-rhamnopyranosyloxy)benzyl-ITC	*Reseda odorata*	2.0	10	1.4	4.0	6.8
*6*	benzyl-ITC	*M*. *rivae*	4.0	—	—	—	—
*7*	1-methylethyl-ITC	*M*. *peregrina*	>10	—	—	—	—
–^d^	4α(methylsulfinyl)butyl-ITC [sulforaphane]	*Brassica oleracea*	0.2	1.6	0.2	1.0	1.5

^**a**^**GS** – glucosinolate (see Fig. 1) from which ITC was produced (experimental details in text) following addition of purified myrosinase to the incubation mixtures. None of the GS were inducers in native form but had to be converted to their isothiocyanate congeners. ^**b**^**NQO1** & ^**c**^**GSH** refer to CDs (the CD is the µM concentration required to double activity following 2-day induction of **NQO1** (NAD(P)H:quinone oxidoreductase 1) in **Hepa**1c1c7, RAW 264.7, **PE**, and **ARPE-**19 cells, or concentration of glutathione (**GSH**) following 1-day induction in **Hepa**1c1c7 cells, respectively; ^d^These ITCs were produced in similar fashion as those from *Moringa* spp., from the GS isolated from the species indicated in the “Source” column (not *Moringa* spp.), and are only included as a point of reference.

**Figure 2:**
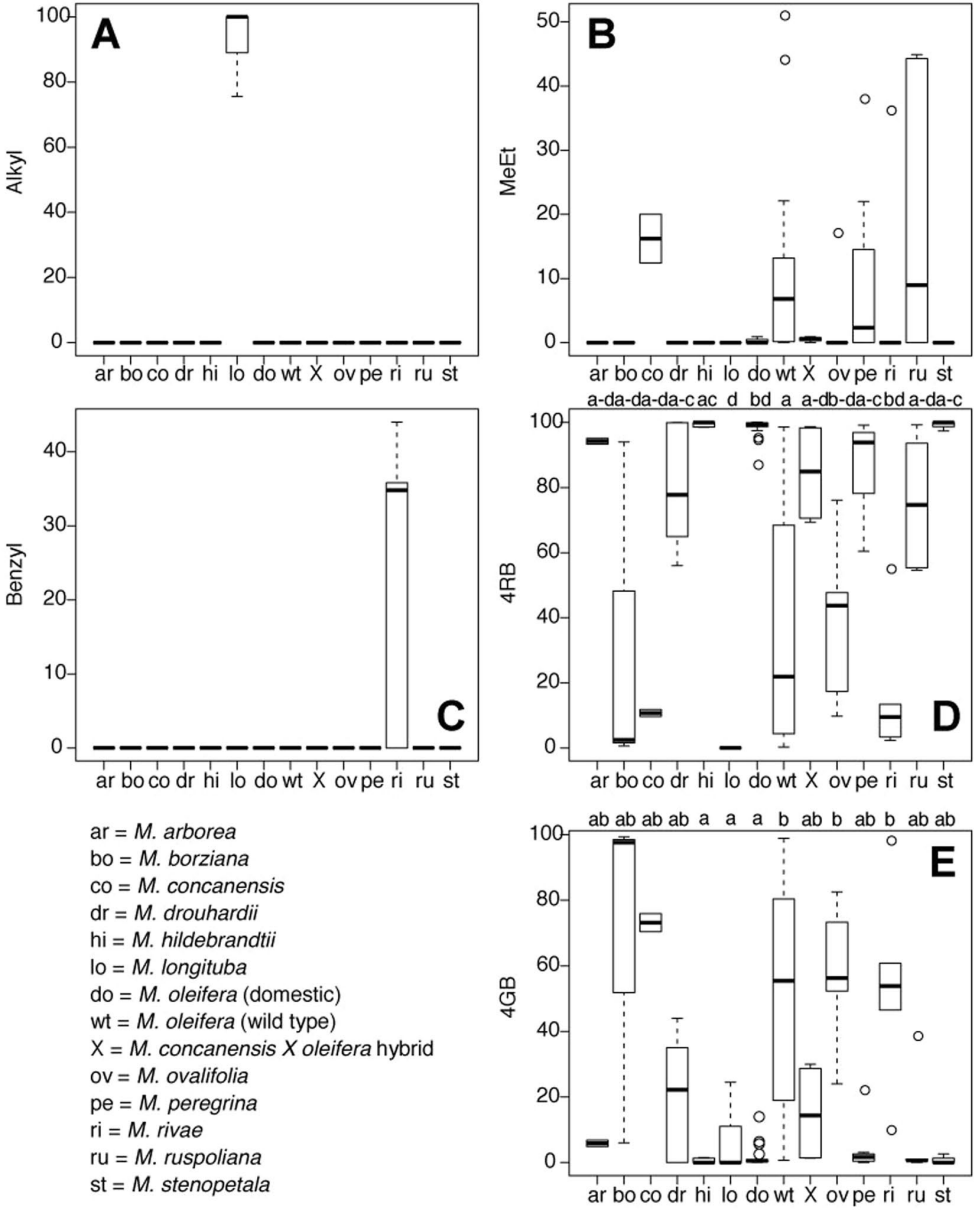
Differences in percentages of the glucosinolates (GS), by glucosinolate, across leaf samples of all species (species designations indicated in the lower left panel of this Figure). (**A**) Alkyl-GSs (1 or more of 5 different isomeric GSs not represented in Fig. 1 –3-hydroxybutyl GS, 4-hydroxybutyl GS, 1-(hydroxymethyl)propyl GS, 2-hydroxy-2-methylpropyl GS, and 1-ethyl-2-hydroxyethyl GS); (**B**) Methylethyl-GS (compound ***7***); (**C**) Benzyl-GS (compound ***6***); (**D**) 4RB (compounds ***1*** and ***2*** combined); (**E**) 4GB (compounds ***3*** and ***4*** combined). Different letters at the tops of **2D** and **2E** indicate statistically homogeneous groups identified by Kruskal-Wallis test followed by post-hoc comparisons.

**Figure 3:**
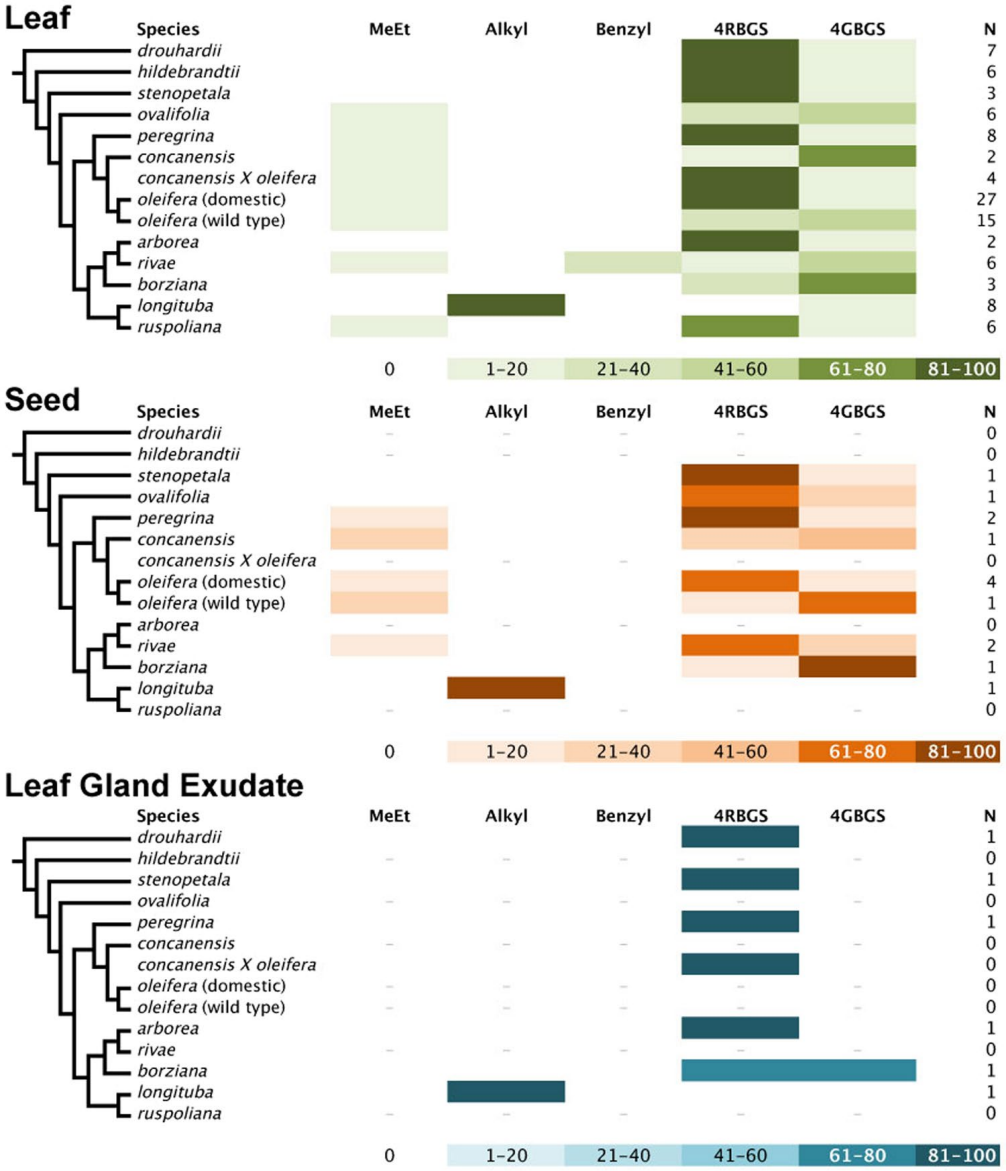
The 13 known species of *Moringa* (family Moringaceae) are shown on the left, with a cladogram showing their relationships. Bars are color coded to represent approximate relative amount of each glucosinolate (GS)(sample size “N” given at right), in the cases of leaves and seeds, and only very small fresh leaf exudate collections. Differences in shading intensity corresponds to percentages of glucosinolates. GSs are grouped along structural lines in some cases, thus: **MeEt** (compound **7**); **Alkyl** (one or more of 5 different isomeric GSs not represented in Fig. 1: 3-hydroxybutyl GS, 4-hydroxybutyl GS, 1-(hydroxymethyl)propyl GS, 2-hydroxy-2-methylpropyl GS, and 1-ethyl-2-hydroxyethyl GS); **Benzyl** (compound **6**); ***4RBGS**** (compounds* **1** and **2**); **4GBGS** (**3** and **4**). Note that source material for *M*. *rivae* included shoot apices.

Detailed comparisons of silica gel dried and fresh leaves in *M*. *oleifera* found that silica gel drying satisfactorily preserved GS levels and myrosinase activity^61^. We therefore treated silica gel and fresh leaf samples as equivalent and pooled them in subsequent analyses. We then asked whether young leaves, which had not finished expanding and were largely non-lignified, differed in their glucosinolate levels from mature leaves, which had ceased expansion and were more lignified. We also found no significant differences, though p-values were often marginal, suggesting that more detailed analysis might detect different profiles between mature and immature leaves (Table 2). Finally, we observed significant differences in at least one species with respect to the others for each of the GS (Table 2). These differences across species are most obvious in the leaves, of which we were able to secure the most extensive collection of plant material, allowing us to make some strong comparisons between species and across GS (Fig. 2). Though sampling was relatively limited, our results suggest major quantitative differences between plant organs in their GS content and spectrum. Leaves differ both quantitatively and qualitatively in their GS profiles from seeds (9 species compared), and from leaf gland exudates (7 species compared) (Fig. 3, Supplemental Table S1).

**Table 2:** Wilcoxon signed-rank tests for differences in glucosinolate levels between silica-gel dried and fresh, and mature and immature leaves, and Kruskal-Wallis tests for differences in glucosinolate levels between species.

Glucosinolate	Dried vs. fresh	Mature vs immature	Between species
Met	V = 5, p = 1.0	V = 15, p = 0.06	χ^2^_13_ = 44.12, p < 0.0001
Alkyl	V = 1, p = 1.0	V = 1, p = 1.0	χ^2^_13_ = 101.87, p < 0.0001
Benzyl	na^a^	V = 0, p = 1.0	χ^2^_13_ = 66.59, p < 0.0001
4RB (glucomoringin)	V = 13, p = 0.93	V = 9, p = 0.07	χ^2^_13_ = 75.85, p < 0.0001
4GB (glucosoonjnain)	V = 16, p = 0.84	V = 38, p = 0.70	χ^2^_13_ = 62.27, p < 0.0001
^b^N	8	11	14

^a^na- not included in dried vs fresh comparison because we included mature leaves only, and only observed in *M*. *rivae* immature leaves.

^b^N the sample size reflects the number of taxa included in the test (species plus wild/domestic *M*. *oleifera* and the *M*. *concanensis* X *oleifera* hybrid).

**Figure 4:**
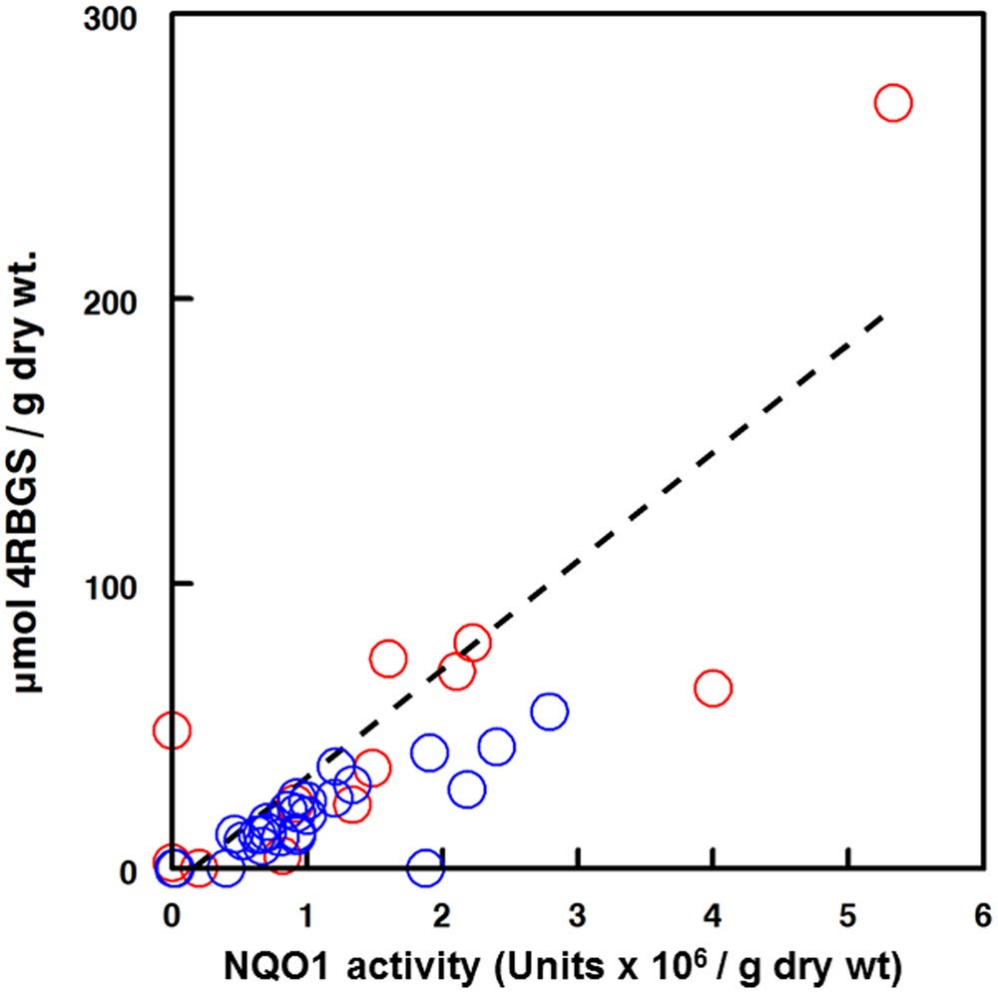
NQO1 inducer potential strongly correlated with 4RBGS content. Single extracts of representative leaves from each of the 12 Moringa species were assayed by the “Prochaska assay”^47,85^ with added myrosinase to convert glucosinolates (GS) to their biologically active cognate isothiocyanates, and to then determine relative potency as an inducer of the phase 2 cytoprotective response. Aliquots of the same samples were evaluated by HPLC to determine concentration of 4RBGS ***1***. Results are reported as NQO1 inducer potential (million Units). Both dry (; blue) and fresh (; red) leaf samples were assayed, however the latter were normalized to a dry weight basis by assuming 75% moisture content, in order to overlay data. (The mean moisture content of experimentally measured values ranged from 70 to 80%, with a mean of 75%). Potency was highly correlated with 4RBGS content (Pearson’s r = 0.825 for the overall regression).

Overall, cytoprotective enzyme inducer potency for 11 of 12 *Moringa* leaf extracts was comparable to that observed for broccoli seeds, which are the most potent plant source of this activity^62^. Potency was highly correlated with the plants’ content of 4RBGS ***1*** on both a dry and a fresh weight basis (Pearson’s *r* = 0.850 and 0.844 respectively) (Fig. 4). *Moringa* *longituba* was unique among the species examined in that it contained predominantly the very simple alkyl side-chain GS as already described in this section, none of which are very potent inducers of the Keap1-Nrf2-ARE mediated cytoprotective enzymes, typified by NAD(P)H:quinone oxidoreductase 1 (NQO1) when assayed separately (*data not shown*). Interestingly, it also contained no detectable 4RBGS.

**Figure 5:**
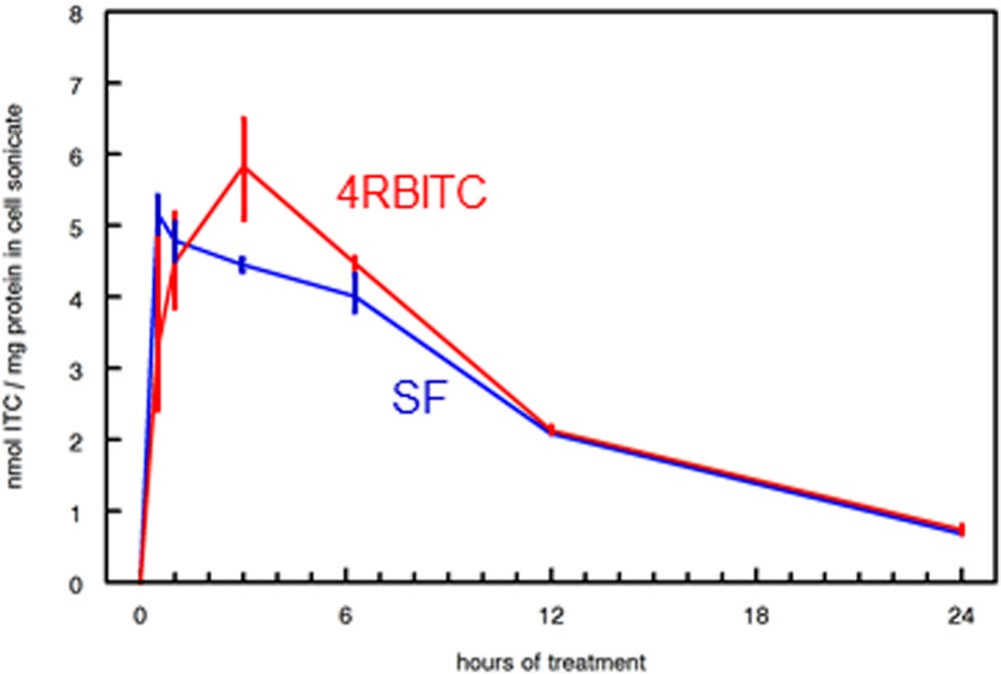
Pharmacokinetics of uptake of 4RBITC (produced from compound ***1***) and sulforaphane (Inducer potency of SF is similar whether produced *in situ* by myrosinase, or purchased as either R- [natural] or RS- [synthetic] SF added to culture medium for Hepa1c1c7 murine hepatoma cells). Area-under-the-curve (AUC) is very similar for the two compounds, 57.77 for SF and 62.62 for 4RBITC, but 4RBITC does not reach its maximum level in cells until 4 hours, whereas SF has already peaked by 1 hour post-addition. 4RBITC is about 10-fold more potent an inducer in this cell line. The difference in uptake kinetics may be reflected in greater potency. Values for duplicate determinations at each time point are connected by solid vertical lines.

Intrigued by the almost 10-fold stronger NQO1 induction in Hepa1c1c7 cells by 4RBITC compared to sulforaphane (4-methylsulfinylbutyl-ITC), which comes from broccoli and is not present in *Moringa* spp., uptake of the ITCs 4RBITC and sulforaphane was compared in a number of cell lines. A representative uptake curve in cultured Hepa 1c1c7 cells is presented (Fig. 5), in which kinetics were similar, and “area under the curve” (AUC; a commonly used pharmacokinetic metric) was essentially the same as that produced by equimolar quantities of 4RBITC, making it appear that factors beyond differences in uptake must account for the large difference in potency between 4RBITC and sulforaphane.

## Discussion

Our results document very wide variation in GS profiles and potency of induction of mammalian phase 2 cytoprotective enzymes across *Moringa* species. These results illustrate the importance of examining as wide an array as possible of germplasm, both domesticated and wild, because the variation across species greatly exceeded that within the cultivated domestic variants of *M*. *oleifera*, by far the most commonly cultivated and readily obtainable species. Underscoring this diversity, our survey highlights two heretofore unrecognized GS, one very abundant in the putative wild ancestor to *M*. *oleifera*. We discuss this diversity and show how variation in GS profiles is of great potential applied importance, with the potency of phase 2 enzyme induction varying far beyond that known of *M*. *oleifera*, the only species studied to date. We identified a species with much higher phase 2 enzyme induction potential than *M*. *oleifera*, as well as species with no or very low phase 2 response. We conclude by emphasizing the urgency of conserving all of the species in this genus, not just the relatives of the domesticated *M*. *oleifera*.

### A New Glucosinolate within *M*. *oleifera*

Sampling widely across the family highlighted the presence of heretofore unrecognized glucosinolates. One of these compounds, 4-(-L-glucopyranosyloxy)benzyl GS (4GBGS), is very abundant in the wild type of *M*. *oleifera*, and because we first found it in this species, we name it after this taxon. The most abundant glucosinolate in *M*. *oleifera* is known as glucomoringin. Our name employs a parallel construction based on the common name of the wild type of *M*. *oleifera* in its area of natural occurrence. Though the putative wild type of *M*. *oleifera* is grown widely across eastern Pakistan and lowland northern India from Haryana, Punjab, and Jammu and Kashmir east to Bihar [Garima, Olson, and Nouman unpublished observation], it is native only to a small area. This area is found only on the Punjab-Himachal Pradesh border in northwestern India where the most frequent common name for the plant is “soonjna.” We assign the common name for this glucosinolate as “glucosoonjnain.” The common name for the isothiocyanate resulting from its hydrolysis would thus be “soonjnain”.

Previous surveys of glucosinolates in *M*. *oleifera*^63^ did not find evidence of glucosoonjnain because they all examined domestic, rather than wild type material. Our detailed survey of 4GBGS and 4RBGS across domestic and wild type accessions of *M*. *oleifera*^61^ showed marked differences in profiles. While both glucosinolates are present in both species, 4GBGS is present in very low amounts in domestic *M*. *oleifera* but is the most abundant one in wild type *M*. *oleifera*. The opposite is true of 4RBGS, which is present in very low amounts in wild type *M*. *oleifera* but is the most abundant one in domestic *M*. *oleifera*. Because the two variants differ markedly in taste^61^, it is possible that these dramatically different profiles between the putative wild ancestor and the domesticate are due to selection favoring more agreeable taste. That a novel glucosinolate was waiting to be discovered in the best-known species, *M*. *oleifera*, emphasizes the importance of examining as diverse an array as possible of samples^62,64^, both within and across species.

In addition to 4RBGS, first documented by Badgett^65^ and later reported by others^60^ the following other GS have been reported to occur in *M*. *oleifera*: 4″-acetyl-4-RBGS^66^, benzyl GS^66^; isopropyl- or methylethyl GS, 2-methylpropyl GS, isobutyl GS^20,59,60,67^; and more recently 4-(2′-*O*-acetyl-α-L-rhamnosyloxy)benzyl ITC^68,69^, 4-(4′-*O*-acetyl-α-L-rhamnosyloxy)benzyl ITC^43^; 2′-*O*-acetyl-α-L-rhamnosyloxybenzyl ITC^70^; 3′-*O*-acetyl-α-L- rhamnosyloxy)benzyl ITC^70^; 4′-*O*-acetyl-α-L-rhamnosyloxy)benzyl ITC^70^. Note that nomenclature of the acetylated derivatives is inconsistent but the most often reported of these compounds is 4′-*O*-acetyl-4-(α-L-rhamnopyranosyloxy)benzyl GS (***2***).

### Glucosinolate Diversity Across Species

In addition to novel GSs, our results highlight remarkable variation across species in their glucosinolate profiles (the relative abundances of the different GS). The well-known domestic form of *M*. *oleifera* had as its predominant GS the relatively well-characterized 4RBGS (“glucomoringin”), as did species such as *M*. *arborea*, *M*. *hildebrandtii*, and *M*. *stenopetala*. Other species, including the wild type of *M*. *oleifera*, *M*. *concanensis*, *M*. *longituba*, and *M*. *rivae*, had dependably low levels of 4RBGS. *Moringa longituba*, a dwarf species from Kenya, Ethiopia, and Somalia with a slender aboveground stem springing from a large belowground tuber, appears to have no or trivial amounts of 4RBGS, unique in the family. The newly-documented 4GBGS (“glucosoonjnain”) varied conspicuously across species, and was especially prominent in accessions of *M*. *ovalifolia* and *M*. *rivae*, and in wild type *M*. *oleifera*. 4RBGS and 4GBGS were present in almost all species, and the remarkable variation in the levels of these GS across accessions and across species strongly suggests that the levels of these compounds is malleable under selection, giving promise to efforts to shape the levels of these GSs under breeding programs.

In contrast to 4RBGS and 4GBGS, which were widespread across the genus, the other main categories of GS were observed only in certain species. The most widespread of these was 1-methylethyl-GS (compound ***7***, Fig. 1). Absent from most species, this compound was observed in *M*. *ruspoliana*, a species with very large leaflets from the Horn of Africa, as well as *M*. *peregrina*, a species with minute, deciduous leaflets found in the Arabian Peninsula and adjacent areas. Although levels of methylethyl-GS were consistently very low in domestic *M*. *oleifera*, it was often present in appreciable proportions in the wild species from the Indian subcontinent, *M*. *concanensis* and *M*. *oleifera* wild type. More remarkably, compound ***6***, benzyl-GS, was only observed to any appreciable degree in young leaves of *Moringa rivae*, a poorly-studied shrubby species from remote drylands of Kenya, Ethiopia, and Somalia. In addition to benzyl GS, *M*. *rivae* also had appreciable levels of 4RBGS and 4GBGS. 4RBGS and 4GBGS were nearly absent from *M*. *longituba*, as were all other GS except for the alkyl GS. The alkyl GS, in turn, were absent in all other species. With both structural variation and variation in relative proportions across species, our results highlight the vast variation potentially available for breeding across the small number of taxa studied.

### Glucosinolates Differ with Ontogeny

In addition to variation across species, our data suggest that GS profiles may vary between mature and immature leaves. We did not, however, detect statistically significant differences between mature and immature leaves in the proportions of any of the GS structural categories (Table 2). In the case of methylethyl-GS and 4RBGS, the p-values were nearly significant. Since GS are favored in herbivore defense in poorly lignified ontogenetic stages, it seems plausible that more detailed study will recover significant differences between immature and mature leaves. Inspection of the patterns in our data (Fig. 3; Supplemental Table S1) suggest that in most species (*M*. *arborea*, *M*. *borziana*, *M*. *drouhardii*, *M*. *hildebrandtii*, *M*. *oleifera* domestic, *M*. *peregrina*, *M*. *ruspoliana*, and *M*. *stenopetala*), 4RBGS was the predominant GS in young leaves. Only in *M*. *longituba*, *M*. *ovalifolia*, *M*. *rivae* were GS other than 4RBGS predominant in young leaves. Our sampling of immature leaves included very small leaves still undergoing some cell division, as well as much larger ones mostly undergoing cell expansion and cell wall lignification. These observations closely parallel our observations in *Brassica oleracea* var. *italica* (broccoli) in which GS profile changes dramatically in ontogeny^10,71^, with genetics^72,73^, and environment^71,73^. This heterogeneity across the ontogenetic stages sampled here means that there is opportunity for more detailed examination of the ways that GS profiles vary from emergence to maturity in leaves of mature trees, as well as how GS vary in *Moringa* from seedlings to saplings.

### Glucosinolates Differ Across Organs

Just as different ontogenetic stages of the same organ could be expected to differ in glucosinolate profiles, organs with different roles can also differ, and our results are suggestive in this regard. Seeds (n = 9) were the non-leaf organ for which we had the most data. Inspection of Supplemental Table S2 suggests that the profile of GS in seeds generally parallels that of those in mature leaves. Leaf gland exudate was available from 6 species, and in contrast to seeds, exudates differed from leaves in their GS profiles. Glucosinolate diversity was always very low in the exudates, usually limited to a single GS with the exception of *M*. *borziana*, which bore two. The GS present in exudate were always the predominant GS in the leaves from a species. *Moringa* *arborea*, *M*. *drouhardii*, *M*. *peregrina*, and *M*. *stenopetala* exudates had as their only glucosinolate 4RBGS, which was the predominant GS in leaves (and seed, when available). Similarly, exudate in *M*. *longituba* contained only alkyl glucosinolate(s), which are also predominant in *M*. *longituba* leaf and seed profiles. Only in *M*. *borziana* leaf exudates were two GS (4RBGS and 4GBGS) observed. While 4RBGS predominated in the leaves of the plants of the same provenance as the leaf gland exudate, the proportions were nearly equal in the leaf gland exudate. Finally, we collected bark from a *M*. *longituba* population and from *M*. *arborea* because the plants were leafless at the time of our field visit. The bark sample of *M*. *longituba* is noteworthy because it was the only sample of that species that had appreciable levels of 4RBGS. The inflorescence axis of *M*. *arborea* had slightly higher levels of 4GBGS than the other samples but otherwise paralleled the leaf and exudate profiles. Though the sample sizes were small, our results comparing leaf glucosinolate profiles with other plant parts are sufficient to suggest strongly that exploring not only across ontogenetic stages but also across plant organs is very likely to recover biologically significant variation in glucosinolate profiles.

### Glucosinolates and Phylogeny

There was no tendency for the GS profiles of closely related species, or species of similar habit or habitat to resemble one another more than GS profiles of species that were distantly related or dissimilar in habit or habitat. Perhaps most notably, the domestic *M*. *oleifera* differed strongly from the wild type in having high levels of 4RBGS and low levels of 4GBGS. Like wild type *M*. *oleifera*, the other wild Indian species, *M*. *concanensis*, also had much higher levels of 4GBGS than the domestic, an example of presumably close relationship not predicting similarity of GS profiles. Another striking example is provided by *M*. *longituba* and *M*. *ruspoliana*, which are sister species, i.e. sharing a most recent common ancestor and each other’s closest living relative. They share morphological features from wood anatomy to flower color and leaf texture. However, their GS profiles were maximally different, with alkyl GS, abundant in *M*. *longituba*, not detected in *M*. *ruspoliana*, and methylethyl GS, which are relatively abundant in *M*. *ruspoliana*, entirely absent from *M*. *longituba*. 4RBGS, which is abundant in *M*. *ruspoliana* leaves, was observed only in leaf gland exudate in *M*. *longituba*.

A major advantage often cited for phylogenetic classifications is that they are predictive. Predictive in this sense means that phylogenetic affinity should predict similarity in characteristics such as morphology or phytochemistry. While this is surely true at the ordinal level—*Moringa* species are more similar to one another in their GS profiles in general than they are to members of any other family in the order—it is not so within the family. It is essentially impossible to predict the GS profile of one species given its phylogenetic position and the profiles of the other species. This marked variation in important biologically active compounds strongly underscores the need both to study comprehensively the diversity in the family as well as the need to conserve the species. With no way of predicting the GSs present, there is no way of knowing what is lost if any taxa or populations become extinct before they are studied.

### Glucosinolate Structure and Function

Comparing the rhamnopyranosyloxylbenzyl- bearing GSs in *Moringa* with those in *Reseda*, the only other plants known to produce such GSs, provides clues regarding the molecular structural features that are correlated with biological activity in *Moringa*. *Moringa oleifera* is of interest because its leaves have high levels of 4RBGS, a GS with a unique rhamosyloxy residue in the side chain. Myrosinase hydrolysis of 4RBGS leads to a highly chemoprotective ITC, such that in certain cell types it is a more potent inducer of the prototypical phase 2 enzyme response than sulforaphane from broccoli sprouts, the most potent naturally occuring phase 2 inducer known to date^31,37^. *Moringa arborea*, a poorly known species that has only been seen twice by botanists, induced an extremely high cytoprotective response, and was correspondingly rich in 4RBGS. It seems plausible that structural variation in the rhamnosyloxy moiety could affect functional aspects such as lipophilicity, solubility, and bioavailability^47^.

We examined the functional impact of variation in the position of the rhamnosyloxy moiety by measuring the phase 2 induction capacity of Compound ***5***, which is from *Reseda odorata* and is not known from *Moringa*. As compared to 4RBGS, Compound ***5*** showed 40-fold lower induction capacity in Hepa1c1c7 cells and 4-fold lower in RAW264.7 cells, did not appreciably change it in PE or ARPE-19 cells, and actually increased by 3-fold its ability to induce glutathione synthesis in Hepa1c1c7 cells (Table 1). 4RBGS and Compound ***5*** are isomers that differ only in the position of the rhamnosyl group on the side-chain benzyl ring. In 4RBGS, the rhamnosyl group is in the the *para*- (or C4- position; see Compound ***1*** in Fig. 1), whereas it is at the *ortho*- (or C2- position) in Compound ***5***; Fig. 1). This positional difference is associated in most cases with dramatically lower chemoprotective potency of Compound ***5*** as compared to 4RBGS. The *meta*- or 3-substituted isomer has to our knowledge never been identified in the plant kingdom or elsewhere, but examining its activity if found or synthesized is a priority. With regard to the activities of other *Moringa* compounds, neither the alkyl GS ***7***, nor the aryl GS ***6*** (Fig. 1) were very potent inducers of cytoprotective enzymes. Likewise, the new “glucosoonjnain” (compound ***3***), is 20-, 2-, 3-, and 3-fold less potent than the rhamnosylated congener (compound ***1***) as an inducer of the cytoprotective enzyme NQO1 in Hepa1c1c7, RAW 264.7, PE, and ARPE-19 cells, respectively. Though the mechanism remains to be elucidated, our findings implicate the rhamnosoxyl group and its position on the benzyl ring as strongly affecting biological activity.

How best to ingest *Moringa* to maximize the health benefits deriving from its GSs has been little studied, but it is likely to be similar to other GSs, for which abundant information is available. Myrosinase, as with most other enzymes, is irreversibly inactivated by the heat of cooking^3,74–76^, and so the co-administration of myrosinase can potentially offset some of the lack of myrosinase in cooked vegetables^77^. As a result, consuming *Moringa* leaves with minimal heat, either raw or only barely cooked, would seem to maximize chances of receiving biologically significant doses of ITC. Crushing of leaves before consumption is also expected to raise ITC levels significantly. Though most of these observations have not been replicated with the GS-myrosinase-ITC system in moringa, it is extraordinarily unlikely that these observations would not also apply to this genus.

## Conclusions

We must now start to better understand not only why these compounds are produced by plants (for their defense, as both anti-feedants and attractants), but how and why different relative quantities are made, what cues changes in these ratios, and most importantly, how each of them works to alter human healthspan when ingested or applied topically. Much work has been done clinically with broccoli sprouts and sulforaphane, but 4RBITC is even more potent than sulforaphane in some assays (Table 1, and refs^15,25,47^). Further understanding of the biological properties of these compounds, and of their pharmacokinetics and pharmacodynamics in human beings, will permit more rational and targeted prescriptive and personalized nutrition – “green chemoprotection” or “frugal medicine”^37,38^. Identification of *Moringa* GS has with few exceptions been limited to the most commonly cultivated species *M*. *oleifera* and *M*. *stenopetala*. Knowledge of the unique GS and powerful chemoprotective activity from members of this genus should bolster efforts to prevent the impending extinction of these invaluable additions to our medical and nutritional armamentarium^78^.

## Materials and Methods

### Plant Collection

Samples of 12 of the 13 species of *Moringa* were collected in the field or obtained from cultivated specimens. Pressed, dried herbarium specimens were prepared as vouchers for most collections. Most of the species grow in remote and difficult to access localities, so this is the first time that material of so many species has been assembled for study of the glucosinolate diversity in the group. This rarity means that some species were available only in very limited quantities. For example, the collections here of *M*. *arborea* represent only the second time the plant has ever been seen by scientists. With regard to *M*. *pygmaea*, this species is native to a very remote area of the Somaliland-Puntland border^79^ and samples were not available. The plants sampled and locality information are listed in Supplemental Table S2. Voucher specimens are deposited in the Missouri Botanical Garden, Kew, East African, Mexican National, and other herbaria. Samples indicated in Supplemental Table S2 as leaflets dried in silica gel were collected following protocols routinely used for the collection of plant material for DNA extraction^80^. Approximately 10 dried leaflets were ground with a small amount of sterile silica sand. A subsample was used for DNA extraction for phylogeny reconstruction^80^. Some plants were leafless at the time of collection. Therefore, one sample of *M*. *longituba* (Olson 710) was prepared by separating the bark from the xylem cylinder and drying the bark components that remained after separating the phellem. A sample of *M*. *arborea* (Olson 714) was prepared from an inflorescence axis. Samples not collected in the field were from plants grown in the Biology Department greenhouse at Washington University, St. Louis, Missouri, or the International *Moringa* Germplasm Collection in Jalisco, Mexico. Glandular exudates were collected from actively-secreting glands on young leaves with sterile glass capillary tubes. Fresh leaves were also gathered from greenhouse grown plants, packed between moist paper towels, and overnight shipped on ice to Johns Hopkins University (Baltimore, MD, USA). To test ontogenetic differences in GS distribution, we gathered both young and mature leaves. Young leaves were the most apical available and had not completed expansion, whereas leaves considered mature were the lowermost healthy leaves available on the stems, had ceased expansion, and contained more lignified tissue.

### Glucosinolate Extraction

GS extracts were made by homogenizing either dry plant tissues ground with an equal weight of silica sand, or fresh leaves, for 3 minutes in cold solvent (equal parts of acetonitrile, dimethyl sulfoxide, and dimethylformamide)^62^ or in 80% boiling methanol using a Polytron Homogenizer (Brinkman Instruments, Westbury, NY, USA) at ½ speed for 3 min. Homogenates were centrifuged (5600 × *g* for 3 min at 25 °C) and the supernatant was stored at −20 °C until analysis.

### Analysis

Extracts were analyzed by Hydrophilic Interaction Liquid Chromatography (HILIC) according to published methods^81,82^. Briefly, confirmation of GS identities was performed by comparison to analytical standards prepared as described^83^ and by using a complementary protocol for HPLC of intact GS followed by mass spectroscopy^58^. All HPLC was performed with the plant extracts, followed directly by the same extracts after incubation with exogenous myrosinase, which uniquely degrades GS, thus either reducing or eliminating only peaks attributable to GS. Thus, HPLC peaks were judged to be GS only if the spectrum of an isolated peak eluted by HPLC was characteristic of the specific GS, and if repeated chromatography of the extract after hydrolysis with purified myrosinase resulted in a substantial diminution and/or disappearance of the peak. Multiple pooled HPLC peaks were collected for direct injection mass spectroscopy. If, following mass spectroscopy in two separate systems, both absolute mass and MS/MS transitions, were not congruent with known GS, NMR was performed to anchor structural assignments.

### Analysis: Mass Spectroscopy

Confirmatory analysis was performed on all GS, first using mass spectroscopy (MS) in both the positive mode with electrospray ionization tandem MS (ESI-MS/MS using a Thermo-Finnigan TSQ Advantage Triple Quadrupole MS coupled to a Thermo-Finnigan Accela UPLC and HTC Pal autoinjector)^84^ and the negative mode (capillary temperature 320 °C, sheath gas 20 a.u., spray voltage 4 kV)^36^ and with a PE Sciex Q-Star Hybrid Quadrupole/time-of-flight mass spectrometer. With these protocols, masses (m/z), and MS/MS transitions were matched with expectations and with known standards^36,84^. Absolute mass was ultimately obtained for all compounds. Accurate mass analyses were performed by pooling analytical HPLC peaks obtained from sequential injections, concentrating, and introducing directly on an Agilent 6520B quadrupole time-of-flight (QTOF) mass spectrometer in negative mode with drying gas at 350 °C, 11 L/min and 40 psi, in MS mode. This has a mass accuracy of 2 ppm (ca. ± 0.001 amu), and in practice these measurements are usually within ± 0.0003 amu.

### Analysis: NMR Spectroscopy

Proton and carbon NMR data were acquired at 23 °C with a 400 MHz Mercury spectrometer equipped 5 mm z-axis gradient probe. Samples were dissolved in 600 μL of D_2_O at concentrations of 1–10 mM. The observed ^1^H chemical shifts are reported with respect to the residual H_2_O/HOD signals, which is at 4.80 ppm downfield from external sodium 3-(trimethylsilyl) propionate-2-2-3-3-d4 (TSP) in D_2_O. One dimensional ^1^H, ^13^C natural abundance, and two-dimensional ^1^H-^1^H COSY were acquired for each glucosinolate sample.

### Cytoprotective Phase 2 Enzyme Induction Bioassay

Extracts were diluted 200-fold into microtiter plates for bioassay as described^47,62,85^. Briefly, induction of NQO1 catalytic activity was measured in Hepa 1c1c7 murine hepatoma cells grown in microtiter plate wells each containing 150 µL of medium. Eight or more replicates of 1:1 serial dilutions in culture medium were assayed. To measure total inducer activity in plant extracts [from GS and their cognate isothiocyanates], excess purified myrosinase^3,4^ and 500 µM ascorbate to activate the myrosinase were added directly to the microtiter plates. One unit of inducer activity is the amount that doubles NQO1 specific activity in 48 h in a well containing 150 µL of medium. Other cell lines were tested in the same manner. Cell lines were maintained as described previously and reviewed in^47^ and detailed as referenced: Hepa1c1c7 murine hepatoma cells^62^, RAW264.7 murine macrophage cells^86^; PE murine keratinocytes^87^, and APRE-19 human retinal pigment epithelial cells^45^.

### Cellular Uptake Studies

Hepa 1c1c7 cells were seeded at 5 × 10^5^ cells per well in 3 mL culture medium in 6-well (3.5 cm diameter) tissue culture plates for 24 h. Uptake was then monitored over the next 48 h following addition of 10 μM final concentration of each of the isothiocyanates delivered in DMSO (0.1% final concentration). Wells containing ITC but no cells served as blanks; DMSO was added to control wells containing cells only. Culture medium was aspirated and cells were harvested at time points up to 24 h as previously described, using a method designed to rapidly remove all traces of cell culture medium and the ITC contained therein, from cells^88^. Briefly, cells were immediately layered onto a 1:1 mixture of diisononyl phthalate and dibutyl phthalate, centrifuged gently in a swinging bucket centrifuge, chilled, re-suspended in water, stored at −70 °C until assayed, then thawed, sonicated and ITCs were measured by HPLC^14^.

### Reagents

All reagents used were analytical or HPLC grade and were purchased from Sigma/Aldrich Chemical Company, Inc., St. Louis, MO., USA) or from Fisher Scientific (Fairlawn, NJ, USA), USB/Affymetrix (Santa Clara, CA., USA; G6PD), Gibco Life Technologies Corp. (Grand Island, NY, USA; FBS & culture media).

### Data Analysis: Glucosinolate Profiles

Given the uneven distribution of sample sizes across species, we used Wilcoxon signed-rank tests to compare the levels of GS between silica gel dried versus fresh leaf samples, with data paired by species. For these tests, we used mean values of each GS for silica gel dried and fresh leaves per species. Because they represented the bulk of the sample, we used only mature leaves for these tests. We then tested for differences between immature versus mature leaves, using a similar procedure. Finally, we tested for differences between species in each of the GS categories using Kruskal-Wallis tests followed by non-parametric multiple comparisons. Analyses were carried out in R v.3.3.1^89^.

### Ethics Approval

Not applicable since no human or animal studies were conducted herein.

## Electronic supplementary material

Supplementary Information
