# Supplementary material for: The Diversity of Chemoprotective Glucosinolates in Moringaceae (Moringa spp.)
Source: Sci Rep. 2018 May 22;8:7994. doi: 10.1038/s41598-018-26058-4 (PMC5964242; doi:10.1038/s41598-018-26058-4)
Supplement: Supplementary file 1 — Supplementary Information [file 41598_2018_26058_MOESM1_ESM.pdf]

The Diversity of Chemoprotective Glucosinolates in Moringaceae (*Moringa* spp.)

Jed W. Fahey,* Mark E. Olson, Katherine K. Stephenson, Kristina L. Wade, Gwen M. Chodur, David Odee, Wasif Nouman, Michael Massiah, Jesse Alt, Patricia A. Egner, Walter C. Hubbard

*Corresponding author: Dr. Jed William Fahey (jfahey@jhmi.edu), Director, Cullman Chemoprotection Center, 855 N. Wolfe St., Suite 625, Baltimore, Maryland, USA 21205.

Supplemental Data

NMR. Both accurate mass and NMR assignments of 4-(-L-glucopyranosyloxy)benzyl glucosinolate (4GBGS) were consistent with compound ***3*** (**Fig. 1)**, which has not previously been reported. Briefly, NMR connectivities and assignments of the non-exchangeable protons were accomplished using 2D COSY data (see **Supplemental Fig. S1**). The benzyl protons are identified by a doublet (d) at 7.17 belonging to the proton closer to the ester oxygen and a doublet of doublets (dd) at 6.86 ppm. The two sugar moieties are distinguished by their C1-protons, which for the glucosyl group comes at 5.46 and for the pyranosyloxyl (the sugar adjacent to the sulfur atom) comes at 6.94 ppm. For compound ***3***, the pyranosyloxyl C2, C3, C4, C5 and C6 protons are assigned at 3.86, 3.3, 3.72, 1.06 and 2.78 ppm, respectively. The C2, C3, C4, C5 and C6 protons of the glucosyl moiety are 4.03, 3.86, 3.2, 3.0 and 3.42 ppm, respectively. The observation that the two sugar moieties can be distinguished by the distinct chemical shifts of the C1 protons confirms the identity of compound ***3***. The ^{13}C chemical shifts are consistent with the expected values for the various types of carbon atoms. Compound ***4*** was identified using a similar approach.

Supplemental Figure S1. Proton and carbon NMR obtained using a 400 MHz Mercury spectrometer, with one dimensional ^{1}H, ^{13}C natural abundance, and two-dimensional ^{1}H-^{1}H COSY of glucosoonjnain (4-(L-glucopyranosyloxy)benzyl glucosinolate or 4GBGS).

58c

Archive directory: /space/home/adminyl/vnmrsys/data
Sample directory: adminyl_23Feb2006
File: PROTON

Pulse Sequence: s2pul

Solvent: D2O
Ambient temperature
Mercury-400BB "mercury400"

Relax. delay 1.000 sec
Pulse 45.0 degrees
Acq. time 1.999 sec
Width 3194.9 Hz
8 repetitions
OBSERVE H1, 399.7578831 MHz
DATA PROCESSING
FT size 16384
Total time 0 min, 25 sec

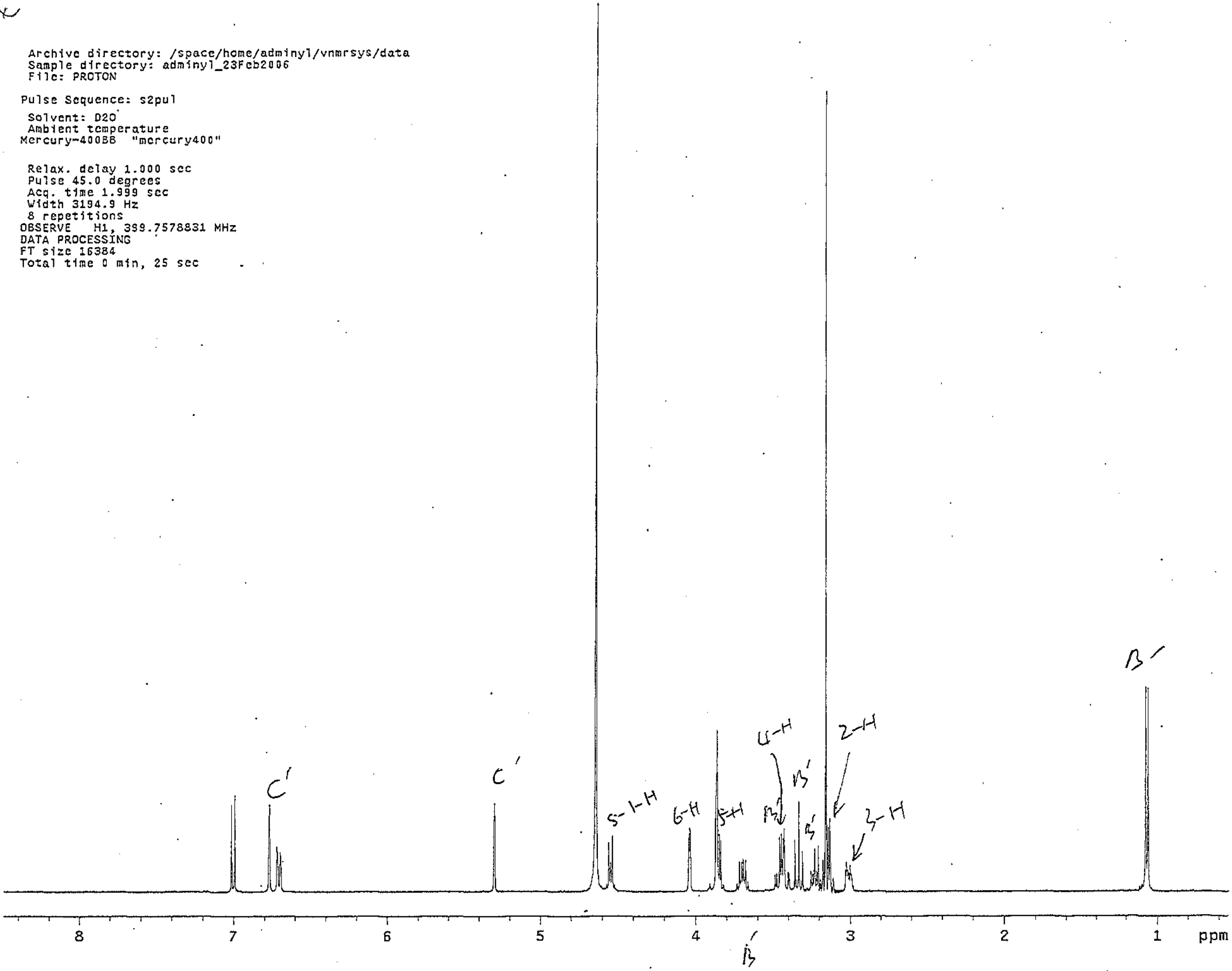

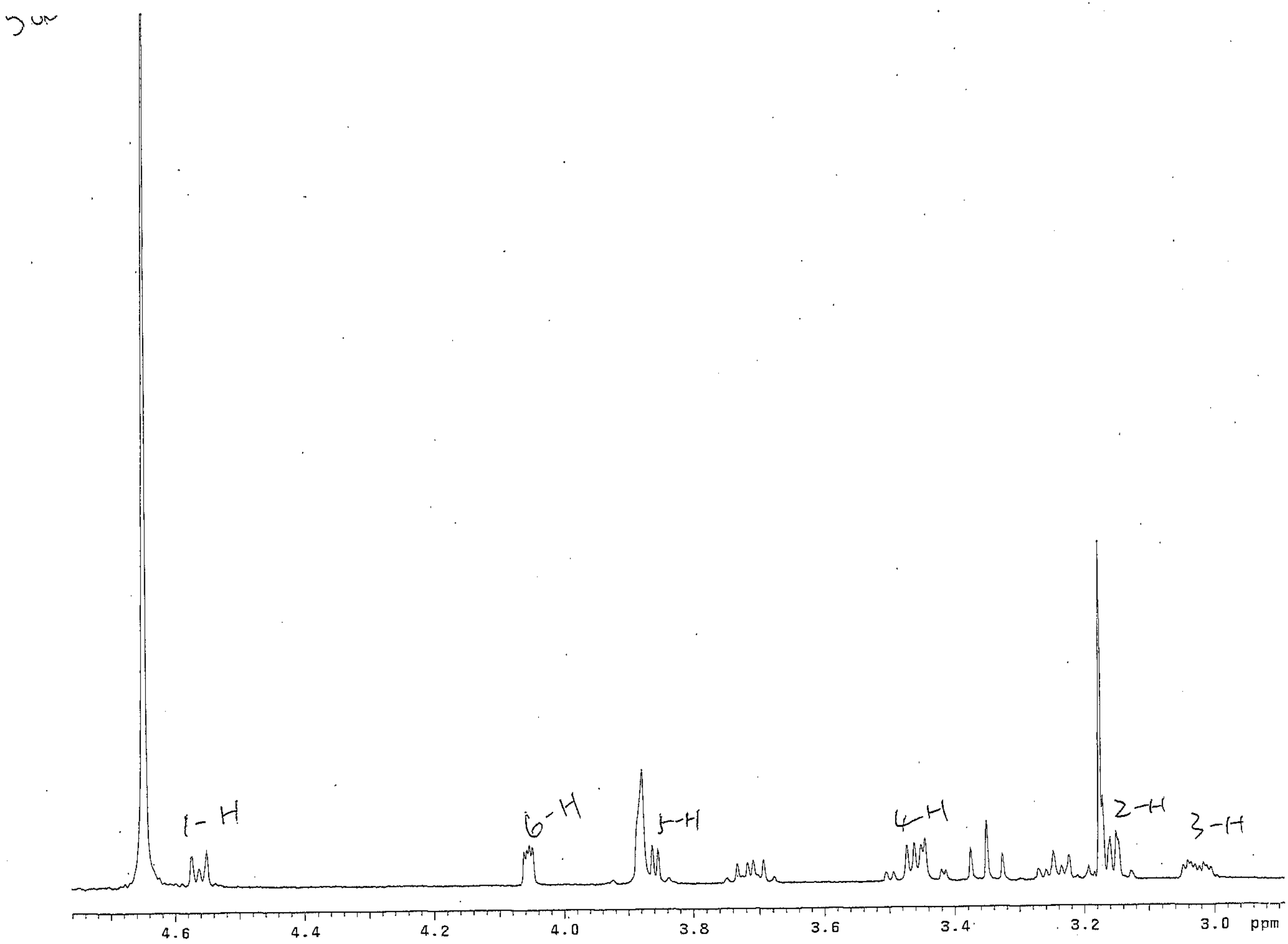
1-H
6-H
5-H
4-H
2-H
3-H
4.6
4.4
4.2
4.0
3.8
3.6
3.4
3.2
3.0
ppm

580

Archive directory: /space/home/adminyl/vnmrsys/data
Sample directory: adminyl_23Feb2006
File: gCOSY

Pulse Sequence: gCOSY

Solvent: D2O
Ambient temperature
Mercury-400BB "mercury400"

Relax. delay 1.000 sec
Acq. time 0.160 sec
Width 3191.8 Hz
2D Width 3191.8 Hz
Single scan
128 increments
OBSERVE H1, 399.7578831 MHz
DATA PROCESSING
Sq. sine bell 0.080 sec
F1 DATA PROCESSING
Sq. sine bell 0.040 sec
FT size 1024 x 1024
Total time 3 min, 37 sec

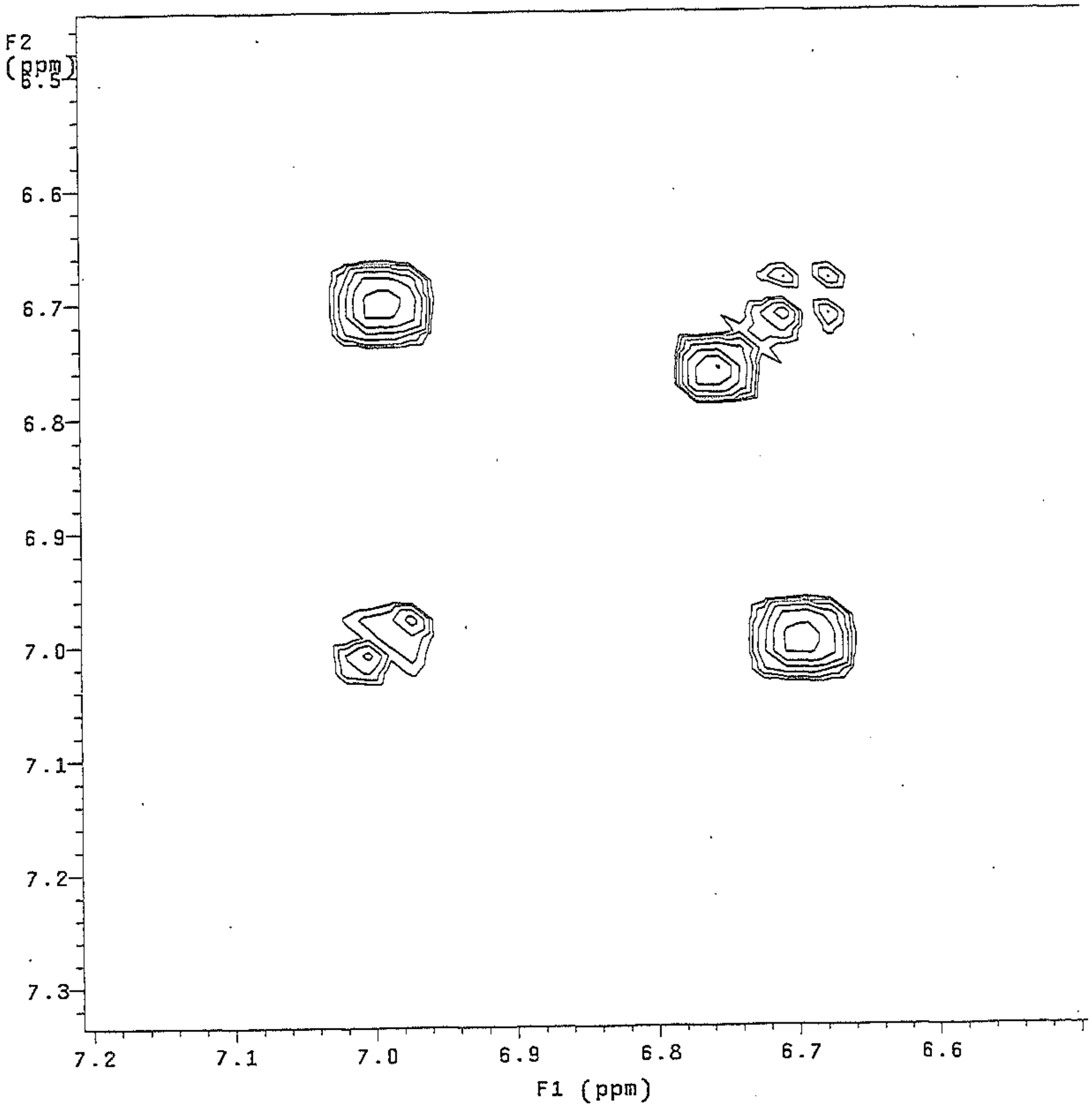

~~486.7~~
586

^{13}C - APT

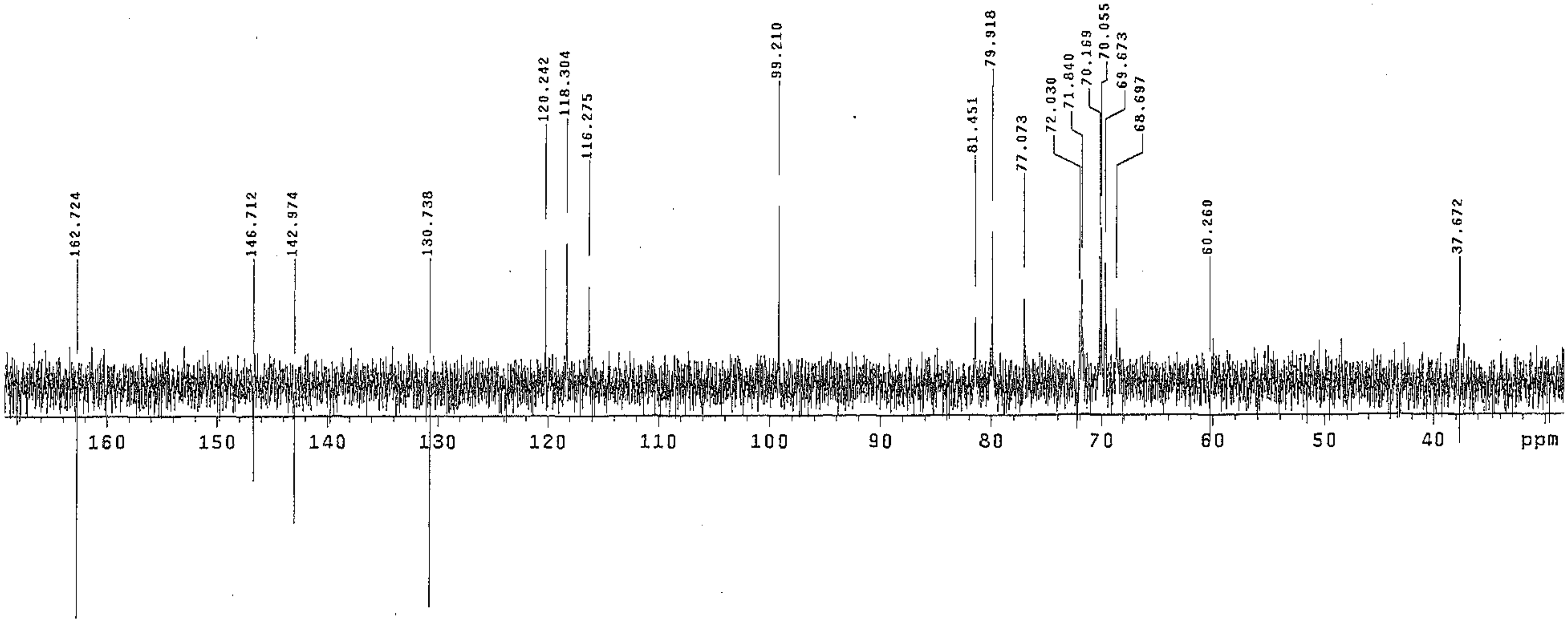

```
exp1  s2pul

      SAMPLE                    SPECIAL
date    Feb 23 2006   temp        not used
solvent         D20   gain        not used
file            exp   spin        not used
   ACQUISITION        hst            0.008
sw           6389.8   pw90          13.700
at            1.998   alfa          20.000
np            25528          FLAGS
fb         not used   il                 n
bs               16   in                 n
d1            1.000   dp                 y
nt                4   hs                nn
ct                4       PROCESSING
   TRANSMITTER        fn          not used
tn               H1        DISPLAY
sfrq        399.760   sp            2138.0
tof       -   437.6   wp             772.2
tpwr             58   rfl           2650.6
pw            6.850   rfp           1918.8
    DECOUPLER         rp              51.2
dn              C13   lp             -81.2
dof               0          PLOT
dm              nnn   wc               250
dmm               c   sc                 0
dpwr             48   vs               200
dmf           17100   th                 4
                      nm   cdc   ph
```

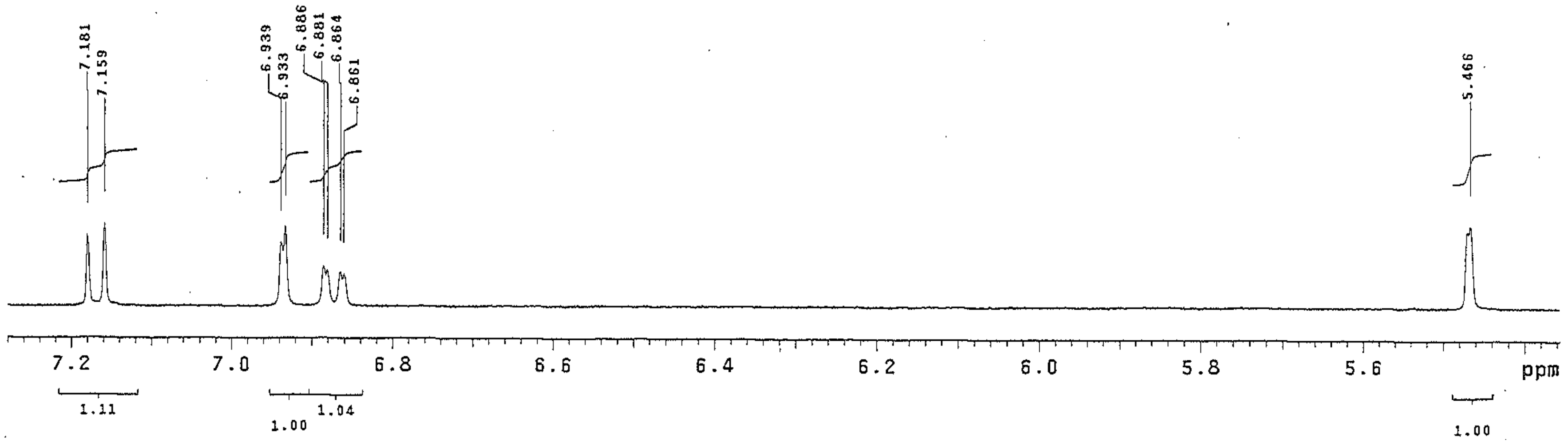

5a

exp1 s2pul

SAMPLE		SPECIAL	
date	Feb 23 2006	temp	not used
solvent	D2O	gain	not used
file	exp	spin	not used
ACQUISITION		hst	0.008
sw	6389.8	pw90	13.700
at	1.998	alfa	20.000
np	25528	FLAGS	
fb	not used	il	n
bs	16	in	n
d1	1.000	dp	y
nt	4	hs	nn
ct	4	PROCESSING	
TRANSMITTER		fn	not used
tn	H1	DISPLAY	
sfrq	399.760	sp	435.2
tof	437.6	wp	2502.0
tpwr	58	rfl	2650.6
pw	6.850	rfp	1918.8
DECOUPLER		rp	51.2
dn	C13	lp	-81.2
dof	0	PLOT	
dm	nnn	wc	250
dmm	c	sc	0
dpwr	48	vs	200
dmf	17100	th	4
		nm cdc ph	

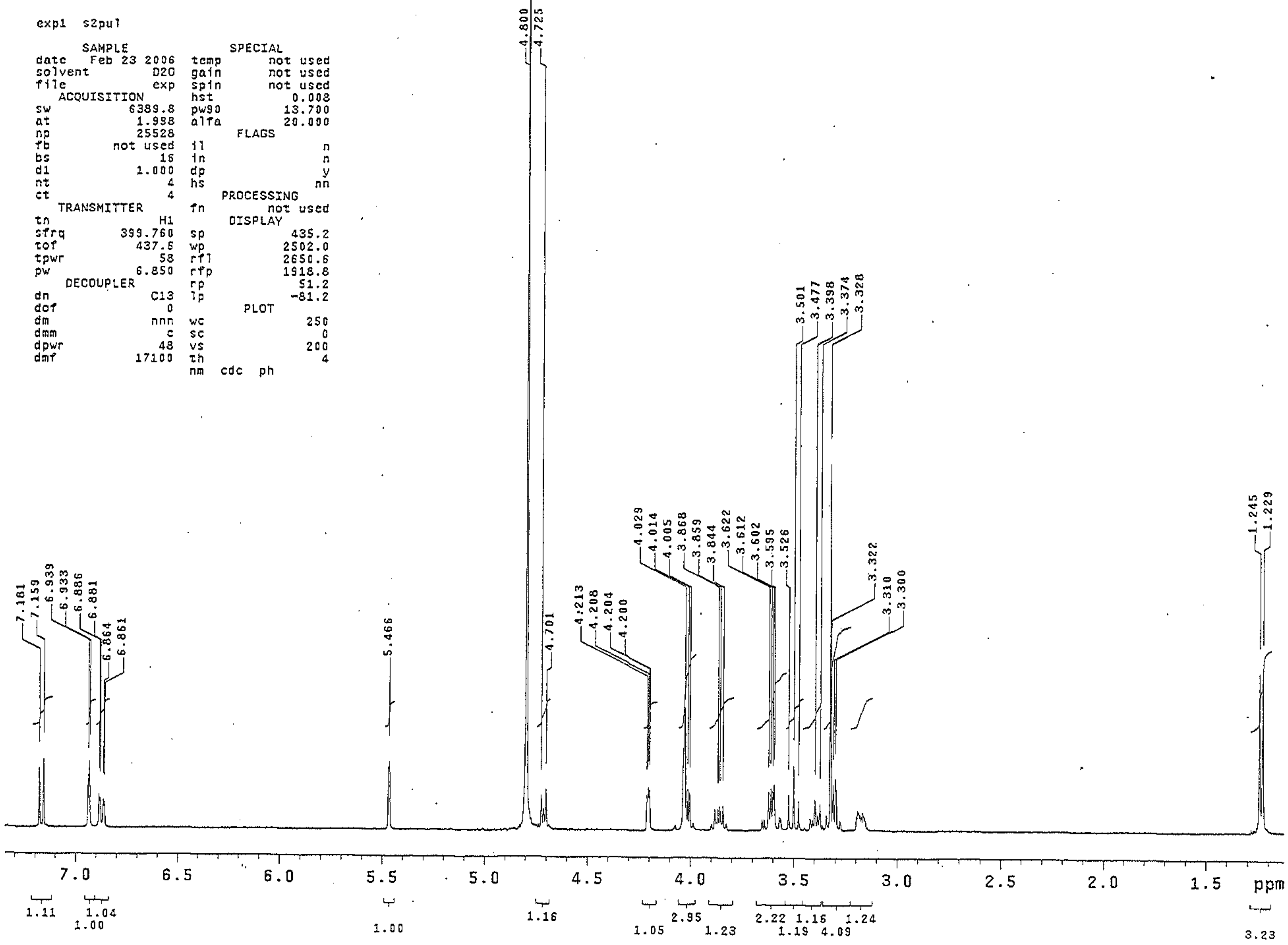

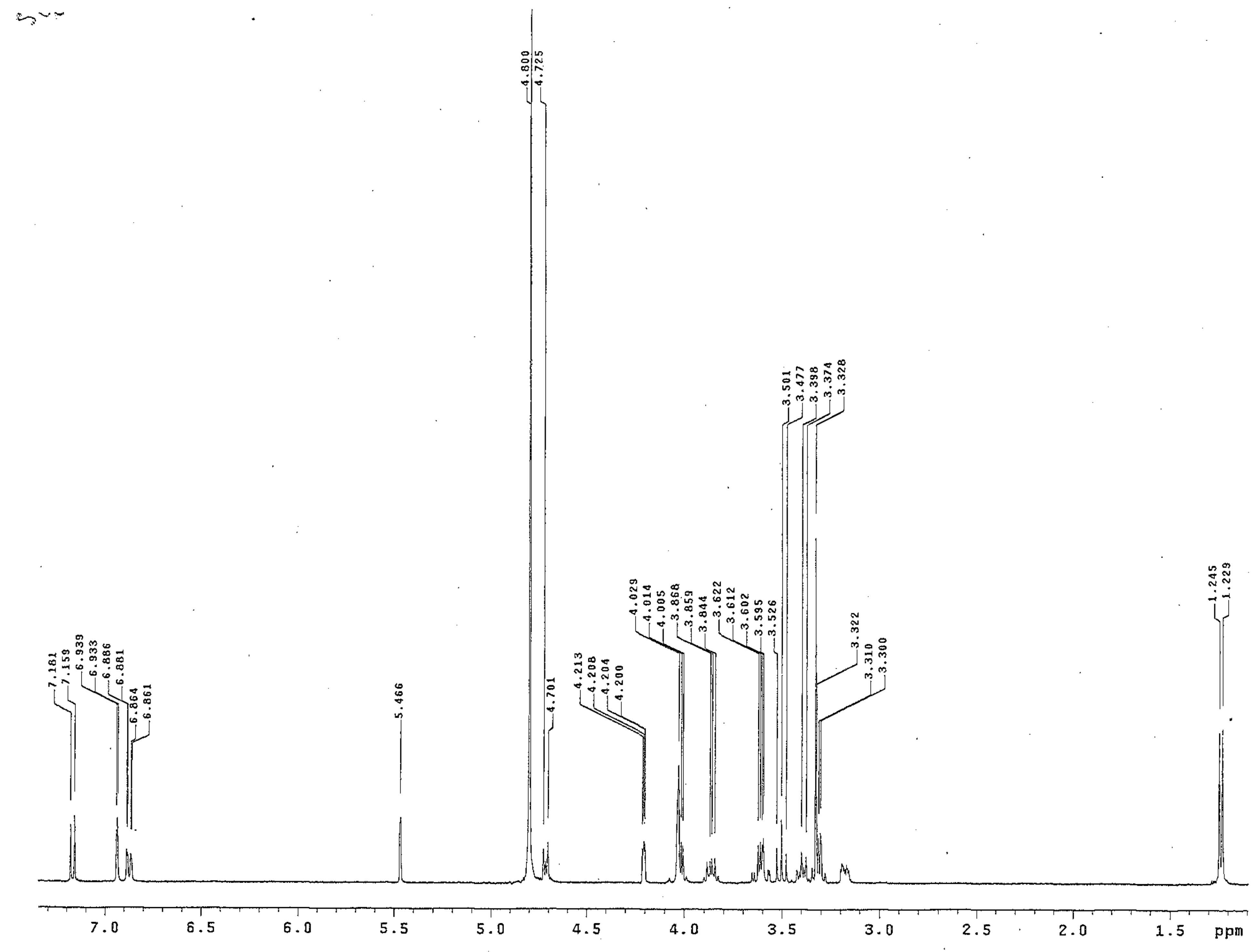

7.181
7.159
6.939
6.933
6.886
6.881
6.864
6.861
5.466
4.800
4.725
4.701
4.213
4.208
4.204
4.200
4.029
4.014
4.005
3.868
3.859
3.844
3.622
3.612
3.602
3.595
3.526
3.501
3.477
3.398
3.374
3.328
3.322
3.310
3.300
1.245
1.229
7.0
6.5
6.0
5.5
5.0
4.5
4.0
3.5
3.0
2.5
2.0
1.5
ppm

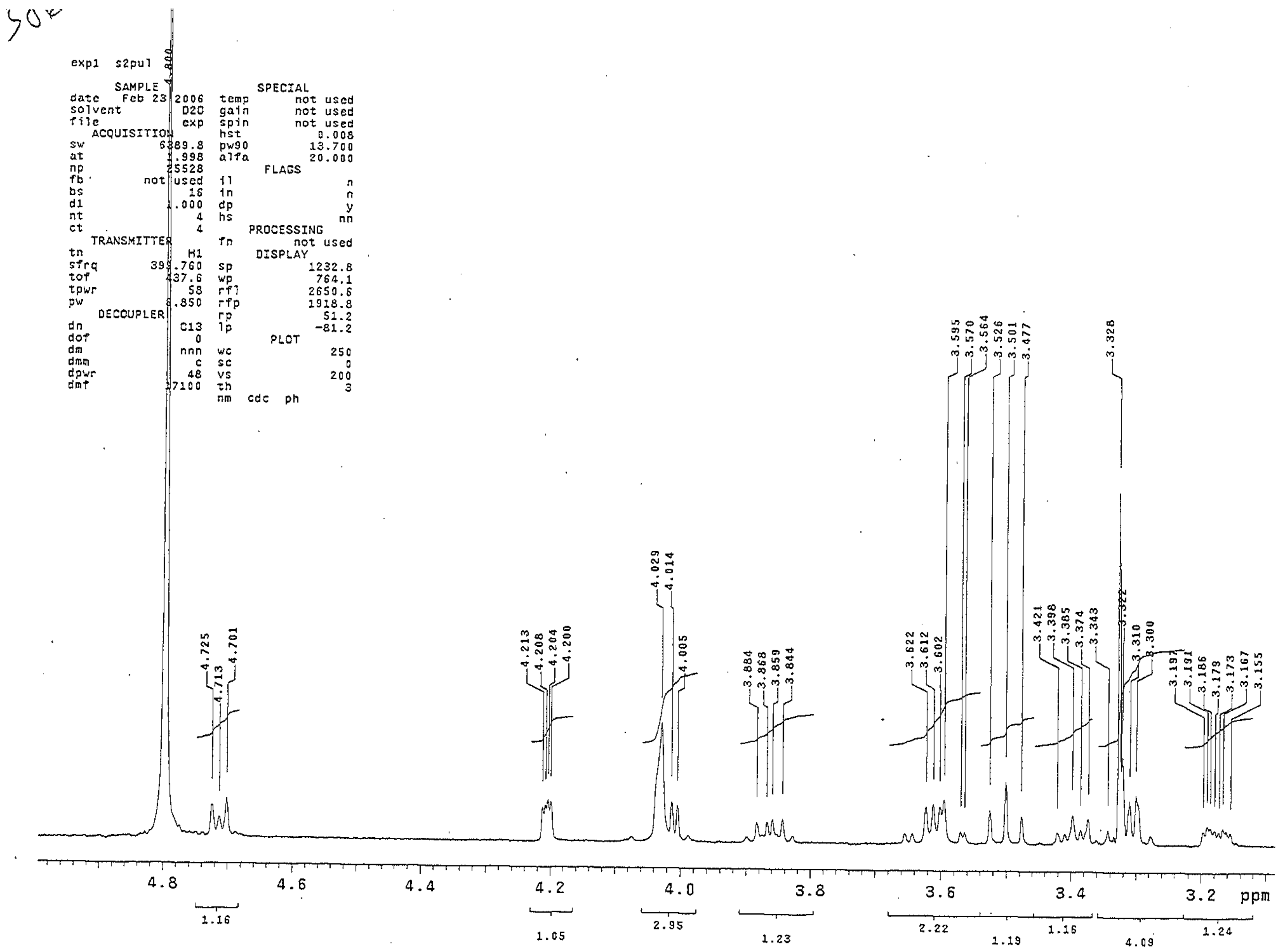
50k
exp1 s2pul
SAMPLE
date Feb 23 2006
solvent D2O
file exp
ACQUISITION
sw 6389.8
at 1.998
np 25528
fb not used
bs 16
d1 1.000
nt 4
ct 4
TRANSMITTER
tn H1
sfrq 399.760
tof 437.6
tpwr 58
pw 6.850
DECOUPLER
dn C13
dof 0
dm nnn
dmm c
dpwr 48
dmf 17100
SPECIAL
temp not used
gain not used
spin not used
hst 0.008
pw90 13.700
alfa 20.000
FLAGS
il n
in n
dp y
hs nn
PROCESSING
fn not used
DISPLAY
sp 1232.8
wp 764.1
rfl 2650.6
rfp 1918.8
rp 51.2
lp -81.2
PLOT
wc 250
sc 0
vs 200
th 3
nm cdc ph
4.800
4.725
4.713
4.701
4.213
4.208
4.204
4.200
4.029
4.014
4.005
3.884
3.868
3.859
3.844
3.622
3.612
3.602
3.595
3.570
3.564
3.526
3.501
3.477
3.421
3.398
3.385
3.374
3.343
3.328
3.322
3.310
3.300
3.197
3.191
3.186
3.179
3.173
3.167
3.155
4.8
4.6
4.4
4.2
4.0
3.8
3.6
3.4
3.2
ppm
1.16
1.05
2.95
1.23
2.22
1.19
1.16
4.09
1.24

58b

exp1 s2pul

SAMPLE		SPECIAL	
date	Feb 23 2006	temp	not used
solvent	D2O	gain	not used
file	exp	spin	not used
ACQUISITION		hst	0.008
sw	25125.6	pw90	13.500
at	1.199	alfa	20.000
np	60270	FLAGS	
fb	13800	il	n
bs	64	in	n
d1	1.000	dp	y
nt	200000	hs	nn
ct	18688	PROCESSING	
TRANSMITTER		lb	1.00
tn	C13	fn	not used
sfrq	100.530	DISPLAY	
tof	1554.7	sp	1201.1
tpwr	63	wp	16013.0
pw	6.750	rfl	1505.7
DECOUPLER		rfp	0
dn	H1	rp	144.4
dof	0	lp	-271.4
dm	yyy	PLOT	
dmm	w	wc	250
dpwr	40	sc	0
dmf	8900	vs	20
		th	5
		nm no ph	

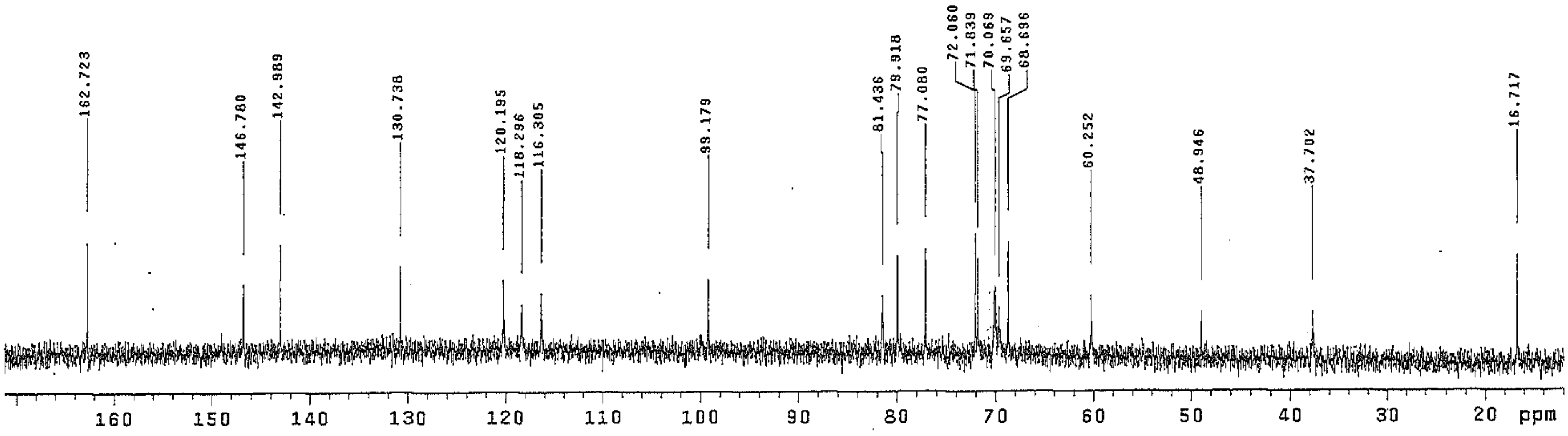

58x.

Archive directory: /space/home/adminyl/vnmrsys/data
Sample directory: adminyl_23Feb2006
File: gCOSY

Pulse Sequence: gCOSY

Solvent: D2O
Ambient temperature
Mercury-400BB "mercury400"

Relax. delay 1.000 sec
Acq. time 0.160 sec
Width 3191.8 Hz
2D Width 3191.8 Hz
Single scan
128 increments
OBSERVE H1, 399.7578831 MHz
DATA PROCESSING
Sq. sine bell 0.080 sec
F1 DATA PROCESSING
Sq. sine bell 0.040 sec
FT size 1024 x 1024
Total time 3 min, 37 sec

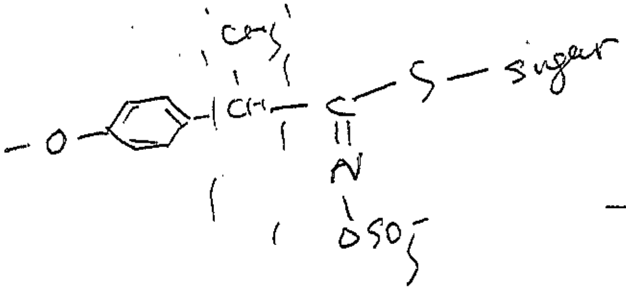

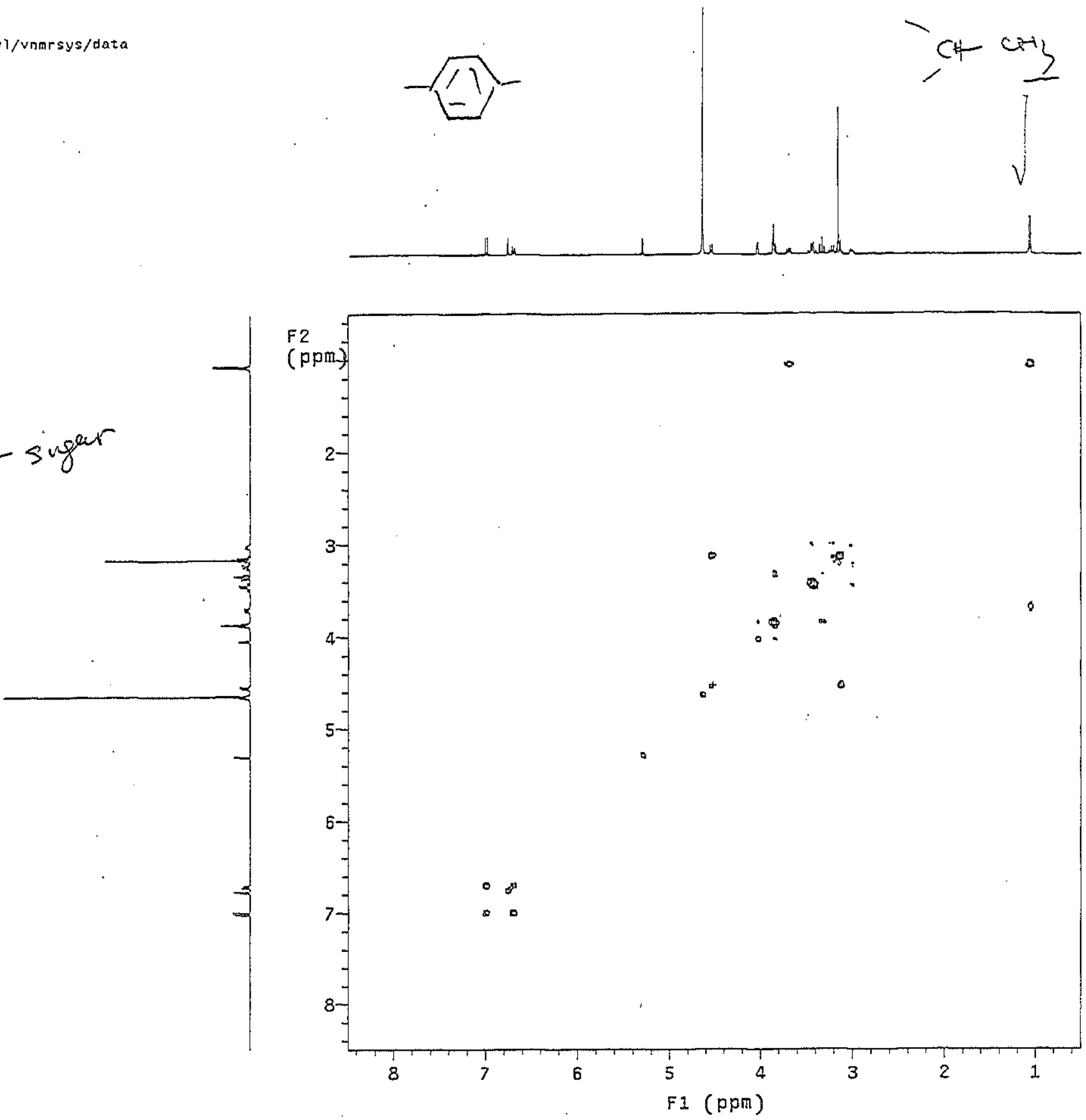

580

Archive directory: /space/home/adminyl/vnmrsys/data
Sample directory: adminyl_23Feb2006
File: gCOSY

Pulse Sequence: gCOSY

Solvent: D2O
Ambient temperature
Mercury-400BB "mercury400"

Relax. delay 1.000 sec
Acq. time 0.160 sec
Width 3191.8 Hz
2D Width 3191.8 Hz
Single scan
128 increments
OBSERVE H1, 399.7578831 MHz
DATA PROCESSING
Sq. sine bell 0.080 sec
F1 DATA PROCESSING
Sq. sine bell 0.040 sec
FT size 1024 x 1024
Total time 3 min, 37 sec

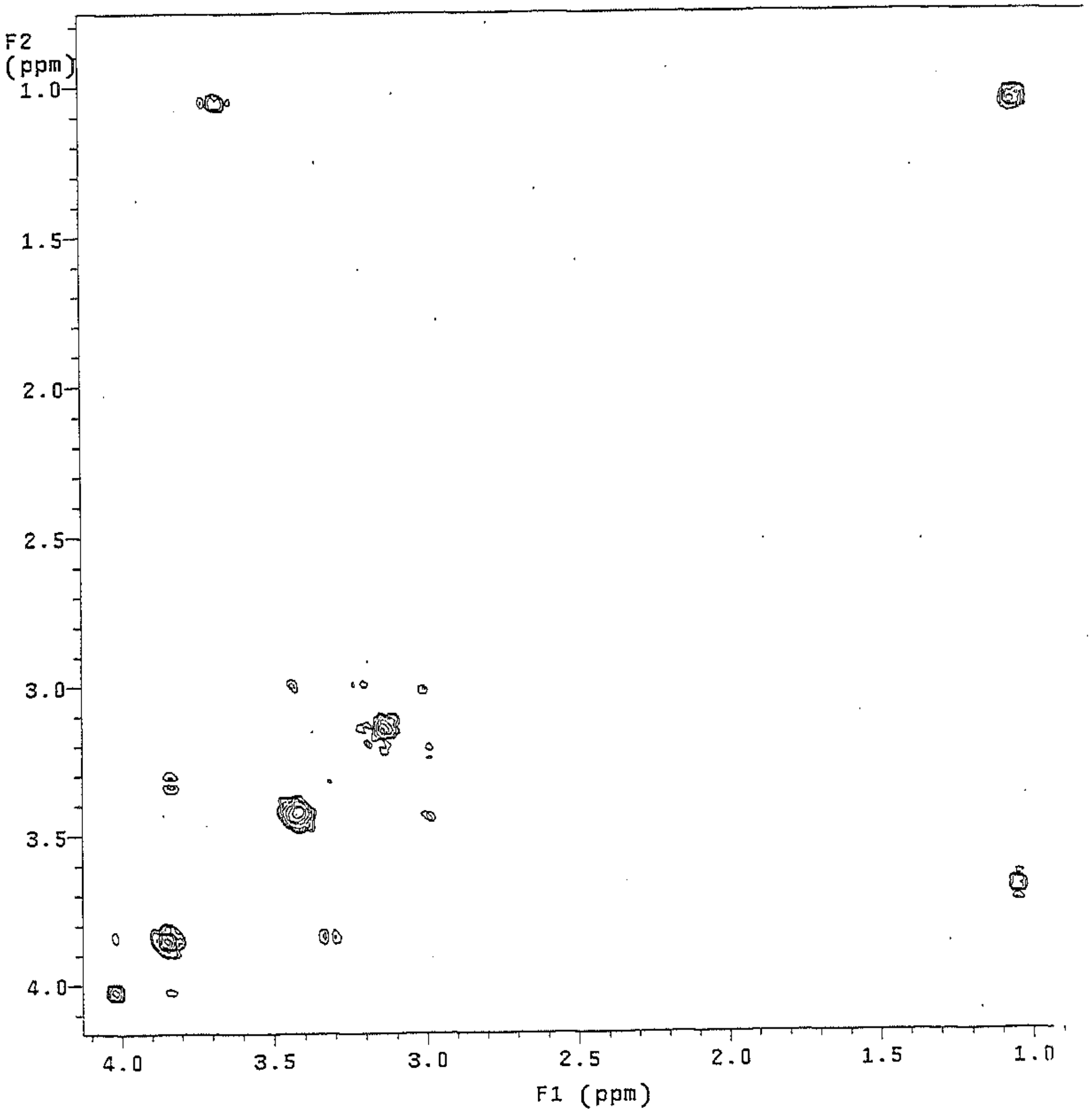

58e

Archive directory: /space/home/adminyl/vnmrsys/data
Sample directory: adminyl_23Feb2006
File: gCOSY

Pulse Sequence: gCOSY

Solvent: D2O
Ambient temperature
Mercury-400BB "mercury400"

Relax. delay 1.000 sec
Acq. time 0.160 sec
Width 3191.8 Hz
2D Width 3191.8 Hz
Single scan
128 increments
OBSERVE H1, 399.7578831 MHz
DATA PROCESSING
Sq. sine bell 0.080 sec
F1 DATA PROCESSING
Sq. sine bell 0.040 sec
FT size 1024 x 1024
Total time 3 min, 37 sec

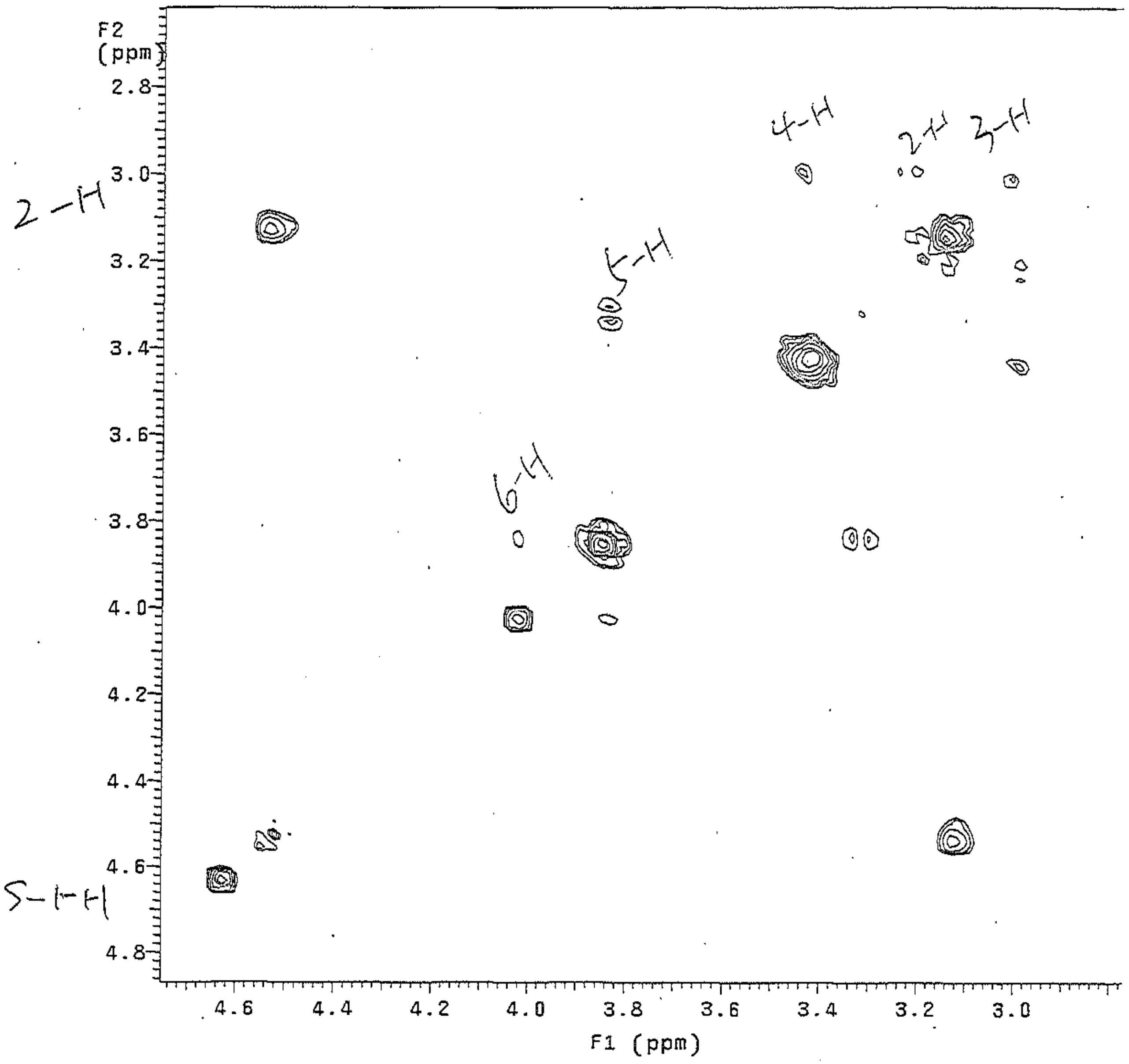

Supplemental Table S1. Means and ranges of percent peak areas of the main glucosinolate categories across the 14 taxa of *Moringa* examined.

Species	Alkyl	Benzyl	4RB (moringin)	4GB (glucosoonjnain)	N
arborea	0	0	94.2 (93.3-95.1)<br>88.8S<br>100E<br>88.8I	5.85 (4.9-6.8)<br>11.2I	2L, 1E, 1I
borziana	0	0	32.37 (0.6-94)<br>2.1S<br>52E	67.67 (6-99.4)<br>97.9S<br>48E	3L, 1S, 1E
concanensis	0	0	10.75 (9.7-11.8)<br>20.7S	73.15 (70.4-75.9)<br>44.2S	2L, 2S
concanensis X *oleifera*	0	0	84.48 (69.4-98.7)	15.03 (1.3-30)	4
drouhardii	0	0	80.51 (56-100)<br>100E	19.49 (0-44)	7L, 1E
hildebrandtii	0	0	99.52 (98.5-100)	0.47 (0-1.5)	6
longituba	94.19 (75.5-100)<br>100S<br>100E<br>95.7B	0	0<br>4.3S	5.81 (0-24.5)	8L, 1S, 1E, 1B
oleifera (domestic)	0	0	98.49 (86.6-100.1)<br>75.23 (3.3-99.4)S	1.32 (0-13.4)<br>17.28 (0.4-67.4)	27L, 4S
oleifera (wild type)	0	0	37.17 (0.3-98.6)<br>0.8S	51.36 (0.7-98.9)<br>62S	15L, 1S
ovalifolia	0	0	39.74 (9.8-76.1)<br>76.2S	57.44 (23.95-82.6)<br>23.9S	6L, 2S
peregrina	0	0	87.18 (60.4-99.2)<br>89.65 (88-91.3)S<br>100E	3.89 (0-21.7)<br>5.55 (2.4-8.7)S	8L, 2S, 1E
rivae	0	24.90 (0-44)	15.46 (2.3-54.6)<br>70.4 (52.2-88.6)S	53.69 (9.5-97.8)<br>29.1 (10.8-47.4)S	6L, 2S
ruspoliana	0	0	75.35 (54.6-99.3)	6.83 (0-38.2)	6
stenopetala	0	0	99.17 (97.4-100.1)<br>99S<br>100E	0.87 (0-2.6)<br>1S	3L, 1S, 1E

Unless indicated, means and ranges refer to leaves. Values designated with the following letters refer to these plant fractions: E=exudate; S=seed; I=inflorescence axis; B=bark

Supplementary Table S2. Provenance of plants or plant organs collected, along with cataloging of glucosinolates found in each of these samples (based on percent of total glucosinolates).

Title: The Diversity of Chemoprotective Glucosinolates in Moringaceae (*Moringa* spp.)

Authors: Jed W. Fahey, Mark E. Olson, Katherine K. Stephenson, Kristina L. Wade, Gwen M. Chodur, David Odee, Wasif Noumann, Michael Massiah, Jesse Alt, Patricia A. Egner, Walter C. Hubbard

							% of total GSs				
Moringa species	Collector	Collection #	Locality	leaf/other	organ type	leaf ontogenetic stage	MeEt	Alkyl	Benzyl	glucomoringin	glucosoonjnain
arborea	Olson	714	Kenya: ca. 35 km NW of Rhamu on road to Malka Mari	inflorescence	inflorescence		0	0	0	88.8	11.2
arborea	Olson	714	Kenya: ca. 35 km NW of Rhamu on road to Malka Mari	leaf	fresh leaf	expanding	0	0	0	95.1	4.9
arborea	Olson	714	Kenya: ca. 35 km NW of Rhamu on road to Malka Mari	leaf	fresh leaf	mature	0	0	0	93.3	6.8
arborea	Olson	714	Kenya: ca. 35 km NW of Rhamu on road to Malka Mari	leaf gland exudate	leaf gland exudate		0	0	0	100	0
borziana	IMGC	375	Kenya: grown from seed provided by Alain Rzepecky, locality unknown	leaf	fresh leaf	expanding	0	0	0	94	6
borziana	IMGC	375	Kenya: grown from seed provided by Alain Rzepecky, locality unknown	leaf gland exudate	leaf gland exudate		0	0	0	52	48
borziana	Olson	678	Kenya: Taita District, ca. 50 m outside Tsavo E National Park Voi Gate	leaf	silica gel dried leaf	mature	0	0	0	0.6	99.4
borziana	Olson	707	Kenya: Kilifi District: Near airstrip at camp on Galana Ranch	leaf	silica gel dried leaf	mature	0	0	0	2.5	97.6
borziana	Olson	5660	Kenya: former Coast Provice: Voi area	seed	seed		0	0	0	2.1	97.9
concanensis	Olson	700	India: Tamil Nadu: Palani Hills near Oddanchatram	leaf	silica gel dried leaf	mature	12.4	0	0	11.8	75.9
concanensis	Olson	700	India: Tamil Nadu: Palani Hills near Oddanchatram	seed	seed		35.2	0	0	20.7	44.2
concanensis	Rao	s.n.	India: Rajasthan: Barmer (collected by S. Rama Rao, Feb 1997)	leaf	silica gel dried leaf	mature	20	0	0	9.7	70.4
drouhardii	Olson	25	USA: Arid Lands nursery, Tucson, Arizona	leaf	fresh leaf	expanding	0	0	0	69.6	30.4
drouhardii	Olson	679	Madagascar: Toliara near Amboasary	leaf	silica gel dried leaf	mature	0	0	0	60.3	39.7
drouhardii	Olson	5707	USA: Hawaii: Koko Crater Botanical Garden	leaf	fresh leaf	expanding	0	0	0	77.8	22.2
drouhardii	Olson	5707	USA: Hawaii: Koko Crater Botanical Garden	leaf	fresh leaf	mature	0	0	0	99.9	0.1
drouhardii	Olson	5708	Madagascar: Toliara: Toliara town	leaf	fresh leaf	mature	0	0	0	56	44
drouhardii	Olson	5708	Madagascar: Toliara: Toliara town	leaf gland exudate	leaf gland exudate		0	0	0	100	0
drouhardii	Olson	679A	Madagascar: Toliara: near Amboasary	leaf	silica gel dried leaf	mature	0	0	0	100	0
drouhardii	Olson	679A	Madagascar: Toliara: near Amboasary	leaf	fresh leaf	expanding	0	0	0	100	0
hildebrandtii	Olson	2	USA: Rare Plant Research nursery, Portland, Oregon	leaf	silica gel dried leaf	mature	0	0	0	98.6	1.3
hildebrandtii	Olson	5709	USA: California: The Huntington	leaf	fresh leaf	expanding	0	0	0	100	0
hildebrandtii	Olson	5709	USA: California: The Huntington	leaf	fresh leaf	mature	0	0	0	100	0
hildebrandtii	Olson	E	USA: Arid Lands nursery, Tucson, Arizona	leaf	fresh leaf	mature	0	0	0	100	0
hildebrandtii	Olson	E	USA: Arid Lands nursery, Tucson, Arizona	leaf	fresh leaf	post-primordia	0	0	0	100	0
hildebrandtii	Olson	E	USA: Arid Lands nursery, Tucson, Arizona	leaf	silica gel dried leaf	mature	0	0	0	98.5	1.5
longituba	Horwood	s.n.	(former) Somalia: Las Anod	leaf	silica gel dried leaf	mature	0	100	0	0	0
longituba	IMGC	36	Puntland: plant provided by Ernst Specks, locality unkown	leaf gland exudate	leaf gland exudate		0	100	0	0	0
longituba	IMGC	36	Puntland: plant provided by Ernst Specks, locality unkown	seed	seed		0	100	0	0	0
longituba	Olson	1	Ethiopia: cultivated plant from Out of Africa nursery	leaf	silica gel dried leaf	mature	0	100	0	0	0
longituba	Olson	704	Kenya: Ca. 13 km WNW of Mandera town on road skirting south bank of the Dawa River, near th	leaf	silica gel dried leaf	mature	0	100	0	0	0
longituba	Olson	704	Kenya: former Northeastern Province: Mandera District: c. 20 km WNW of Mandera near locality	leaf	fresh leaf	expanding	0	78	0	0	22
longituba	Olson	704	Kenya: former Northeastern Province: Mandera District: c. 20 km WNW of Mandera near locality	leaf	fresh leaf	mature	0	75.5	0	0	24.5
longituba	Olson	709	Kenya: Wajir: Lafaley village 8 km N of Wajir	leaf	silica gel dried leaf	mature	0	100	0	0	0
longituba	Olson	712	Kenya: Wajir: 20-35 km E of Wajir on road to Wajir Bor	leaf	silica gel dried leaf	mature	0	100	0	0	0
longituba	Olson	710a	Kenya: Wajir: 20-35 km E of Wajir on road to Wajir Bor	leaf	silica gel dried leaf	mature	0	100	0	0	0
longituba	Olson	710a	Kenya: Wajir: 20-35 km E of Wajir on road to Wajir Bor	bark	silica gel dried bark		0	95.7	0	4.3	0
oleifera (dome	Fahey	2	India: Tamil Nadu: Purchased from Horti Nurseries	leaf	silica gel dried leaf	mature	0	0	0	99.3	0.7
oleifera (dome	Fahey	3	US Virgin Islands: St Croix	seed	seed		0	0	0	99.4	0.6
oleifera (dome	Fahey	4	France: Aigues-Vives: purchased from B&T World Seeds	seed	seed		29.4	0	0	3.3	67.4
oleifera (dome	Fahey	5	India: Tamil Nadu: Purchased from Horti Nurseries	seed	seed		0	0	0	99.3	0.7
oleifera (dome	Fahey	6	USA: Florida: Purchased from Educational Concerns for Hunger Organization, Fort Myers	seed	seed		0.7	0	0	98.9	0.4
oleifera (dome	IMGC	1	Madagascar: Anosy: Tolagnaro	leaf	silica gel dried leaf	mature	0	0	0	99.9	0.1
oleifera (dome	IMGC	2	Mexico: Yucatán: Mérida	leaf	silica gel dried leaf	mature	0	0	0	99.9	0.2
oleifera (dome	IMGC	4	Kenya: former Eastern Province: Isiolo	leaf	silica gel dried leaf	mature	0	0	0	99.8	0.2
oleifera (dome	IMGC	6	Thailand: purchased from Superfoods Moringa for Life as PKM cultivar, but has short fruits, unlik	leaf	silica gel dried leaf	mature	0	0	0	99.9	0.2
oleifera (dome	IMGC	9	Kenya: former Eastern Province: Isiolo	leaf	silica gel dried leaf	mature	0	0	0	94.9	5.1
oleifera (dome	IMGC	13	Kenya: former Eastern Province: Isiolo	leaf	silica gel dried leaf	mature	0	0	0	86.6	13.4
oleifera (dome	IMGC	52	Kenya: former Coast Provice: Voi town	leaf	silica gel dried leaf	mature	0	0	0	99.1	0.9
oleifera (dome	IMGC	66	India: Tamil Nadu: Chennai: Chrompet area	leaf	silica gel dried leaf	mature	0.8	0	0	98.4	0.9
oleifera (dome	IMGC	81	USA: New Mexico: grown from seed from Mesa Garden nursery, provenance unknown	leaf	silica gel dried leaf	mature	0.8	0	0	98.6	0.6
oleifera (dome	IMGC	82	France: La Réunion: grown from seed from Le Jardin Naturel nursery	leaf	silica gel dried leaf	mature	0.8	0	0	99	0.2
oleifera (dome	IMGC	85	Germany: Erzhausen: grown from seed from G. Kohres nursery, provenance unknown	leaf	silica gel dried leaf	mature	0.5	0	0	98.8	0.7

oleifera (dome	IMGC	183	India: Tamil Nadu: Chennai area: PKM type cultivar (collected by V. Amalan Stanley July 1998)	leaf	silica gel dried leaf	mature	0.8	0	0	99	0.3
oleifera (dome	IMGC	248	India: Uttarakhand: Purchased from Paritosh Herbals Nursery, Dehra Dun	leaf	silica gel dried leaf	mature	0.9	0	0	98.5	0.6
oleifera (dome	IMGC	249	India: Uttarakhand: Purchased from Paritosh Herbals Nursery, Dehra Dun	leaf	silica gel dried leaf	mature	0.6	0	0	97.5	2
oleifera (dome	IMGC	257	Mexico: Michoacán: Nueva Italia	leaf	silica gel dried leaf	mature	0.5	0	0	99.1	0.4
oleifera (dome	IMGC	288	India: Uttarakhand: Purchased from Paritosh Herbals Nursery, Dehra Dun	leaf	silica gel dried leaf	mature	0	0	0	94.2	5.8
oleifera (dome	IMGC	289	India: Uttarakhand: Purchased from Paritosh Herbals Nursery, Dehra Dun	leaf	silica gel dried leaf	mature	0	0	0	100	0
oleifera (dome	IMGC	299	Mexico: Jalisco: Miguel Hidalgo Nuevo	leaf	silica gel dried leaf	mature	0.2	0	0	99.1	0.65
oleifera (dome	IMGC	324	South Africa: grown from seed from Silverhill Seeds, Cape Town, South Africa	leaf	silica gel dried leaf	mature	0	0	0	100.1	0
oleifera (dome	IMGC	362	Philippines: Batangas: Mabini town	leaf	silica gel dried leaf	mature	0	0	0	99.2	0.9
oleifera (dome	IMGC	363	Philippines: Batangas: Batangas town	leaf	silica gel dried leaf	mature	0	0	0	99.7	0.3
oleifera (dome	Olson	s.n.1	India: Tamil Nadu: Chennai area: PKM type cultivar (collected by V. Amalan Stanley July 1998)	leaf	silica gel dried leaf	mature	0	0	0	99.5	0.5
oleifera (dome	Olson	s.n.2	India: Tamil Nadu: Chennai area: PKM type cultivar (collected by V. Amalan Stanley July 1998)	leaf	fresh leaf	post-primordia	0	0	0	100	0
oleifera (dome	Stanley	s.n.1	India: Tamil Nadu: Chennai: Chrompet area	leaf	fresh leaf	expanding	0	0	0	100	0
oleifera (dome	Stanley	s.n.2	India: Tamil Nadu: Chennai: Chrompet area	leaf	fresh leaf	mature	0	0	0	100	0
oleifera (dome	Stanley	s.n.3	India: Tamil Nadu: Chennai: Chrompet area	leaf	silica gel dried leaf	mature	0	0	0	99.1	0.9
oleifera (wild t	IMGC	17	Pakistan: Punjab: Qila Didar Singh	leaf	silica gel dried leaf	mature	43.9	0	0	0.8	55.4
oleifera (wild t	IMGC	27	Pakistan: Sindh: Khair Pur	leaf	silica gel dried leaf	mature	0	0	0	8.3	91.7
oleifera (wild t	IMGC	57	Pakistan: Sindh: Khair Pur	leaf	silica gel dried leaf	mature	0.2	0	0	38.5	61.3
oleifera (wild t	IMGC	61	Pakistan: Punjab: Qila Didar Singh	leaf	silica gel dried leaf	mature	22.1	0	0	2.6	75.3
oleifera (wild t	IMGC	85	Germany: Erzhausen: grown from seed from G. Kohres nursery, provenance unknown	leaf	silica gel dried leaf	mature	0.8	0	0	98.6	0.7
oleifera (wild t	IMGC	140	India: Chandigarh: purchased from Shankar Nursery, June 2000	leaf	silica gel dried leaf	mature	0	0	0	1.1	98.9
oleifera (wild t	IMGC	141	India: Chandigarh: purchased from Shankar Nursery, June 2000	leaf	silica gel dried leaf	mature	6.8	0	0	0.3	92.9
oleifera (wild t	IMGC	142	India: Chandigarh: purchased from Shankar Nursery, June 2001	leaf	silica gel dried leaf	mature	8.4	0	0	6.1	85.5
oleifera (wild t	IMGC	143	India: Chandigarh: purchased from Shankar Nursery, June 2000	leaf	silica gel dried leaf	mature	50.8	0	0	15.9	33.3
oleifera (wild t	IMGC	152	India: Chandigarh: purchased from Shankar Nursery, June 2000	leaf	silica gel dried leaf	mature	0	0	0	95.4	4.6
oleifera (wild t	IMGC	171	Pakistan: Punjab: Qila Didar Singh	leaf	silica gel dried leaf	mature	11.7	0	0	41.5	46.9
oleifera (wild t	IMGC	192	Pakistan: Sindh: Khair Pur	leaf	silica gel dried leaf	mature	0.1	0	0	95.5	3.5
oleifera (wild t	IMGC	255	India: Chandigarh: purchased from Shankar Nursery, June 2000	leaf	silica gel dried leaf	mature	0.7	0	0	98.2	1.1
oleifera (wild t	IMGC	256	India: Chandigarh: purchased from Shankar Nursery, June 2000	leaf	silica gel dried leaf	mature	14.7	0	0	32.9	52.4
oleifera (wild t	IMGC	279	India: Chandigarh: purchased from Shankar Nursery, June 2000	leaf	silica gel dried leaf	mature	11.2	0	0	21.9	66.9
oleifera (wild t	Olson	5661	India: Chandigarh: purchased from Shankar Nursery, June 2000	seed	seed		37.2	0	0	0.8	62
oleifera x conc	IMGC	11	Mexico: Jalisco: hybrid produced at IMGC	leaf	silica gel dried leaf	mature	0.6	0	0	98	1.5
oleifera x conc	IMGC	22	Mexico: Jalisco: hybrid produced at IMGC	leaf	silica gel dried leaf	mature	0.9	0	0	71.8	27.3
oleifera x conc	IMGC	23	India: Tamil Nadu: Pachalur area (collected by S. Kalaiselvi, July 2001)	leaf	silica gel dried leaf	mature	0	0	0	98.7	1.3
oleifera x conc	IMGC	65	Mexico: Jalisco: hybrid produced at IMGC	leaf	silica gel dried leaf	mature	0.6	0	0	69.4	30
ovalifolia	Olson	716	Namibia: Namib-Naukluft Park: W of Kuiseb Pass	leaf	fresh leaf	mature	16.9	0	0	9.8	73.3
ovalifolia	Olson	718	Namibia: Namib-Naukluft Park: W of Kuiseb Pass	leaf	fresh leaf	mature	0	0	0	17.4	82.6
ovalifolia	Olson	5710	Namibia: Erongo: Karibib	leaf	silica gel dried leaf	mature	0	0	0	44.8	55.2
ovalifolia	Olson	5710	Namibia: Erongo: Karibib	leaf	fresh leaf	mature	0	0	0	76.1	24.0
ovalifolia	Olson	5711	Namibia: Erongo: Karibib	leaf	fresh leaf	post-primordia	0	0	0	42.7	57.3
ovalifolia	Olson	5711	Namibia: Erongo: Karibib	leaf	fresh leaf	mature	0	0	0	47.7	52.3
ovalifolia	Olson	5712	Namibia: National Botanical Research Institute	seed	seed		0	0	0	76.2	23.9
peregrina	Danin	s.n.	Israel: Southern Region: En-Gedi	leaf	silica gel dried leaf	mature	2.1	0	0	94.7	3.1
peregrina	Danin	s.n.	Israel: Southern Region: En-Gedi	leaf	fresh leaf	expanding	0	0	0	99.2	0.8
peregrina	Danin	s.n.	Israel: Southern Region: En-Gedi	leaf	fresh leaf	post-primordia	0	0	0	78.3	21.7
peregrina	Danin	s.n.	Israel: Southern Region: En-Gedi	seed	seed		0	0	0	91.3	8.7
peregrina	Olson	566	Oman: Muscat: Sultan Qaboos University	leaf	silica gel dried leaf	mature	37.8	0	0	60.4	1.9
peregrina	Olson	567	Oman: northern region: Wadi Muaydin 2–3 km N of Birkat Al Mawz	seed	seed		9.6	0	0	88	2.4
peregrina	Olson	568	Oman: ad-Dahiliyah: Wadi Ghul	leaf	silica gel dried leaf	mature	2.6	0	0	95.3	2.1
peregrina	Olson	568	Oman: ad-Dahiliyah: Wadi Ghul	leaf	fresh leaf	mature	7	0	0	93	0
peregrina	Olson	s.n.1	Egypt: grown from seeds purchased in Cairo market, provided by Ben Stern, University of Bradfc	leaf	silica gel dried leaf	mature	0	0	0	98.5	1.5
peregrina	Olson	s.n.2	Egypt: grown from seeds purchased in Cairo market, provided by Ben Stern, University of Bradfc	leaf	fresh leaf	mature	22	0	0	78	0
peregrina	Olson	s.n.3	Egypt: grown from seeds purchased in Cairo market, provided by Ben Stern, University of Bradfc	leaf gland exudate	leaf gland exudate		0	0	0	100	0
rivae	Olson	677	Kenya: Marsabit District: E slope of Mount Baio	leaf	silica gel dried leaf	mature	36	0	0	54.6	9.5
rivae	Olson	701	Kenya: Marsabit District: E slope of Mount Baio	seed	seed		0.5	0	0	52.2	47.4
rivae	Olson	701	Kenya: Rhamu District: W of Rhamu town	leaf	silica gel dried leaf	mature	0	0	0	2.3	97.8
rivae	Samana	1	Kenya: Marsabit District: E slope of Mount Baio	seed	seed		0.6	0	0	88.6	10.8
rivae	Samana	2	Kenya: Marsabit District: locality known as "Soit"; collected by J. Samana 2000	leaf	fresh leaf	post-primordia	0	0	33.9	9.5	56.7
rivae	Samana	3	Kenya: Marsabit District: locality known as "Soit"; collected by J. Samana 2000	leaf	fresh leaf	post-primordia	0	0	35.7	13.4	50.9
rivae	Samana	4	Kenya: Marsabit District: locality known as "Soit"; collected by J. Samana 2000	leaf	fresh leaf	post-primordia	0	0	35.8	3.4	60.8
rivae	Samana	5	Kenya: Marsabit District: locality known as "Soit"; collected by J. Samana 2000	leaf	fresh leaf	post-primordia	0	0	44	9.6	46.5
ruspoliana	Olson	702	Kenya: Mandera District: Near "Border Point One" (the Kenya-Ethiopia-Somalia border point), at	leaf	fresh leaf	mature	0	0	0	99.3	0.7
ruspoliana	Olson	702	Kenya: Rhamu-Dimtu Division, around town of Yabicho, ca. 6.5 km by road WNW of Rhamu.	leaf	silica gel dried leaf	mature	44.9	0	0	54.6	0.5

ruspoliana	Olson	703	Kenya: Mandera District: Near "Border Point One" (the Kenya-Ethiopia-Somalia border point), at	leaf	fresh leaf	mature	12.6	0	0	87.5	0
ruspoliana	Olson	703	Kenya: Mandera District: Near "Border Point One" (the Kenya-Ethiopia-Somalia border point), at	leaf	silica gel dried leaf	mature	44.3	0	0	55.3	0.5
ruspoliana	Olson	703	Kenya: Mandera District: Near "Border Point One" (the Kenya-Ethiopia-Somalia border point), at	leaf	fresh leaf	expanding	5.3	0	0	93.6	1.1
ruspoliana	Olson	705	Kenya: Mandera District: Khalaliyo Division, Ca. 20 km WNW of Mandera town on road skirting s	leaf	silica gel dried leaf	expanding	0	0	0	61.8	38.2
stenopetala	Olson	675	Kenya: Rift Valley Province: Baringo District: Parmalok Island, Lake Baringo	leaf	silica gel dried leaf	mature	0	0	0	97.4	2.6
stenopetala	Olson	675	Kenya: Rift Valley Province: Baringo District: Parmalok Island, Lake Baringo	leaf	fresh leaf	expanding	0	0	0	100.1	0
stenopetala	Olson	675	Kenya: Rift Valley Province: Baringo District: Parmalok Island, Lake Baringo	leaf	fresh leaf	mature	0	0	0	100	0
stenopetala	Olson	675	Kenya: Rift Valley Province: Baringo District: Parmalok Island, Lake Baringo	leaf gland exudate	leaf gland exudate		0	0	0	100	0
stenopetala	Olson	675	Kenya: Rift Valley Province: Baringo District: Parmalok Island, Lake Baringo	seed	seed		0	0	0	99	1
